# Liposomal Bupivacaine in Transversus Abdominis Plane Block for Postoperative Pain Control After Autologous Breast Reconstruction: A Systematic Review and Meta‐Analysis

**DOI:** 10.1002/micr.70126

**Published:** 2025-10-03

**Authors:** Victor F. A. Almeida, Glaudir Donato, Andressa Alves de Carvalho, Wanessa Alves de Carvalho, Ammar Lakda, Yara Dias, Manoela Dantas, Pedro Danielian, Eliana F. R. Duraes

**Affiliations:** ^1^ Department of Anesthesiology Cleveland Clinic Foundation Cleveland Ohio USA; ^2^ Center of Medical Sciences Federal University of Paraíba João Pessoa Brazil; ^3^ Internal Medicine Department Hospital Universitário Lauro Wanderley João Pessoa Brazil; ^4^ Surgery Department Hospital Universitário Lauro Wanderley João Pessoa Brazil; ^5^ Digestive Disease and Surgery Institute Cleveland Clinic Foundation Cleveland Ohio USA; ^6^ Department of Hematology and Oncology Cleveland Clinic Foundation Cleveland Ohio USA; ^7^ Department of Plastic Surgery Cleveland Clinic Foundation Cleveland Ohio USA

**Keywords:** autologous breast reconstruction, liposomal bupivacaine, opioid consumption, pain management, plain bupivacaine, TAP block

## Abstract

**Background:**

Autologous breast reconstruction using abdominally based flaps is common post‐mastectomy, but donor‐site pain often leads to prolonged opioid use. The transversus abdominis plane (TAP) block is a common regional anesthesia technique, with bupivacaine as the standard anesthetic. Liposomal bupivacaine (LB), a prolonged‐release formulation, aims to extend pain relief and reduce opioid consumption, though its efficacy remains debated.

**Objective:**

This systematic review and meta‐analysis compared LB versus plain bupivacaine (PB) in TAP blocks for autologous breast reconstruction, focusing on opioid consumption, pain scores, and hospital stay.

**Methods:**

A systematic search identified randomized controlled trials and observational studies comparing LB (with or without PB) to PB in TAP blocks. Data were pooled using a random‐effects model ( *I* ^2^ ≥ 25%) or fixed‐effects model ( *I* ^2^ < 25%).

**Results:**

Six studies (429 patients) met inclusion criteria. LB was associated with significant reduction in opioid consumption on postoperative days (POD) 1 (MD = −4.99 mg; 95% CI: [−8.42; −1.56], *p* < 0.01, *I* ^2^ = 0%) and POD 2 (MD = −3.35 mg; 95% CI: [−5.74; −0.96], *p* < 0.01, *I* ^2^ = 0%). Pain scores were significantly lower on POD 2 and POD 3. No difference in hospital stay was found (MD = −0.17; 95% CI: [−0.52; 0.18], *p* = 0.34, *I* ^2^ = 83.1%).

**Conclusion:**

LB reduced opioid consumption during the first 48 h postoperatively and modestly improved pain control on POD 2 and POD 3, but did not shorten hospital stay. Further large‐scale RCTs are needed to validate its benefits.

**Abbreviations**
CI: confidence interval
ERAS: Enhanced Recovery After Surgery
LB: liposomal bupivacaine
MD: mean difference
OME: oral morphine equivalents
PB: plain bupivacaine
POD: postoperative day
RCT: randomized controlled trials
SD: standard deviation
TAP: Transversus Abdominis Plane

## 1 Introduction

According to procedural statistics from the American Society of Plastic Surgeons (ASPS), approximately 157,740 breast reconstructions were performed in 2023. Among these, 35,213 were autologous reconstructions, utilizing tissue flaps harvested from the abdomen, back, buttocks, or thigh—representing a 73.25% increase compared to 2015 (American Society of Plastic Surgeons 2023).

Despite the well‐established reconstructive and psychosocial benefits of microsurgical breast reconstruction, effective postoperative pain control remains a significant clinical challenge. Many patients experience moderate to severe discomfort following flap‐based procedures, which can delay recovery, limit early mobilization, and increase reliance on systemic opioids (Kehlet 1997; Knackstedt et al. 2020).

To address these concerns, Enhanced Recovery After Surgery (ERAS) protocols have been increasingly implemented in reconstructive breast surgery (Rendon et al. 2020; Afonso, Oskar, et al. 2017). These multidisciplinary, evidence‐based pathways are designed to minimize postoperative pain, reduce opioid exposure, accelerate return to baseline function, and facilitate earlier discharge without compromising surgical outcomes (Batdorf et al. 2015; Ljungqvist et al. 2017; Sebai et al. 2019). ERAS protocols emphasize multimodal analgesia, opioid‐sparing strategies, early ambulation, and standardized recovery milestones. Studies have shown that ERAS implementation is associated with reduced pain intensity, shorter hospital stays, and a lower incidence of opioid‐related complications (Rendon et al. 2020; Afonso, Oskar, et al. 2017; Batdorf et al. 2015; Ljungqvist et al. 2017; Sebai et al. 2019; Afonso, Newman, et al. 2017).

Regional anesthesia plays a central role in achieving these outcomes. Among the regional anesthesia techniques utilized in abdominal‐based breast reconstruction, the transversus abdominis plane (TAP) block has emerged as a reliable approach to provide site‐specific analgesia by targeting the sensory nerves innervating the anterior abdominal wall, thereby attenuating postoperative pain and reducing opioid consumption (Rendon et al. 2022; Ha et al. 2019; Knackstedt et al. 2024; Nguyen et al. 2024). Bupivacaine, a long‐acting local anesthetic, has been commonly used for this purpose, but its limited duration of action necessitates repeated dosing or continuous infusions (Knackstedt et al. 2024; Nguyen et al. 2024). In response, liposomal bupivacaine (LB) was developed as a prolonged‐release formulation capable of providing pain relief for up to 72 h (Ha et al. 2019; Park et al. 2024; Vyas et al. 2016).

Despite the theoretical advantages of LB, clinical findings regarding its comparative efficacy remain inconsistent. Some studies suggest that the use of LB in TAP blocks significantly reduces opioid consumption in the immediate postoperative period and may be associated with shorter hospital stays (Knackstedt et al. 2024; Jablonka et al. 2017). However, other randomized controlled trials (RCTs) have not demonstrated significant benefits over conventional (plain) bupivacaine (PB) in terms of postoperative analgesia and overall clinical outcomes (Nguyen et al. 2024; Gatherwright et al. 2018). Additionally, the higher cost of LB raises concerns about its cost‐effectiveness, particularly in the context of routine clinical use.

Given this uncertainty, the present study aims to compare the efficacy of LB versus PB in TAP blocks for patients undergoing autologous abdominal‐based breast reconstruction. The primary outcome will be postoperative opioid consumption, while secondary outcomes will include pain scores and hospital length of stay. By synthesizing the available evidence, this study seeks to provide a comprehensive assessment of the clinical benefits and limitations of LB in this patient population.

## 2 Methods

This systematic review and meta‐analysis adhered to the methodologies outlined by the Cochrane Collaboration and followed the PRISMA (Preferred Reporting Items for Systematic Reviews and Meta‐Analyses) guidelines (Higgins et al. 2022; Page et al. 2021).

### 2.1 Eligibility of Included Studies

The inclusion of studies followed the PICOTS criteria (Population, Intervention, Comparison, Outcomes, Timing, and Study Design) with no restrictions on language (Table 1).

**TABLE 1:** PICOTS framework. Eligibility criteria according to PICOTS.

Parameter	Inclusion criteria	Exclusion criteria
Population	Females undergoing breast reconstruction with autologous abdominal flaps	Patients who underwent other surgical procedures
Intervention	TAP block with LB with or without plain PB	Other anesthetics or other routes than the TAP block
Comparison	TAP block with PB alone or continuous PB TAP infusion	Studies that assessed the ERAS protocol in only one study group, studies that did not include PB in the control group, studies in which the control group received blockade strategies other than the TAP block, and studies without a comparator group
Outcomes	Postoperative opioid consumption, postoperative pain, length of hospital stay	Studies not reporting any of these outcomes
Timing	No restrictions on study duration	—
Study design	Observational studies (case–control, cohort), and RCTs	Conference abstracts, case reports and series, letters, books, reviews, and book chapters

Abbreviations: ERAS, Enhanced Recovery After Surgery; LB, liposomal bupivacaine; PB, plain bupivacaine; TAP, Transversus Abdominis Plane.

### 2.2 Search Strategy

Cochrane Library, Embase, PubMed, and Web of Science were consulted to retrieve studies published up to March 19, 2025, that evaluated the effects of combining LB, with or without PB, for TAP block in patients undergoing breast reconstruction with an autologous abdominal flap. The search strategy utilized a combination of keywords, including “bupivacaine”, “breast reconstruction”, “transverse rectus abdominis myocutaneous flap”, and “deep inferior epigastric perforator flap” as outlined in Table S1. This search was supplemented by a review of reference lists of potentially eligible studies and a manual search of key journals in the field of plastic surgery.

### 2.3 Study Selection

Two reviewers (V.F. and A.L.) independently screened titles and abstracts to identify relevant studies. Any discrepancies were resolved through discussion or consultation with a third reviewer (Y.D.). Full‐text articles of the selected studies were then assessed to ensure they met the predefined eligibility criteria. Final inclusion was determined by consensus between the two primary reviewers, with the third reviewer intervening in cases of disagreement.

### 2.4 Data Extraction

Data extraction was conducted independently by two reviewers (V.F. and A.A.C.). Any disagreements were resolved through discussion, with a third reviewer (G.D.) providing a final decision when necessary.

Data collection was performed using a standardized format and was organized in tables and pre‐prepared forms. Extracted information included identification features (first author's last name, year of publication, study design, study period, country), demographic data (study population, subgroups sample size, age, body mass index, comorbidities, smoking history, previous oncology treatment), surgical aspects (laterality, timing, type of abdominal flap, surgery length, complications), intervention aspects (timing and dosage; perioperative protocols), qualitative and quantitative evaluation methods (including follow‐up length and scales applied), and outcomes (postoperative opioid consumption, postoperative pain scores, length of hospital stay).

### 2.5 Endpoints

The primary endpoint was postoperative opioid consumption, evaluated on postoperative days 1 (0–24 h) and 2 (24–48 h). Secondary endpoints included postoperative pain scores on postoperative days 1 (0–24 h) and 3 (48–72 h), assessed using standardized pain scales, length of hospital stay, defined as the number of days from surgery to discharge.

### 2.6 Quality and Bias Assessment

Two independent reviewers (V.F. and Y.D.) assessed the quality of each included study and evaluated the potential for bias using ROB‐2 for RCTs (Sterne et al. 2019) and ROBINS‐I for non‐randomized studies (Jüni et al. 2016). In case of discrepancies in the assessment, a third reviewer (G.D.) was consulted to resolve any disagreements. To assess publication bias, funnel plots were applied. Egger's test was not performed due to the limited number of studies included in the analysis.

### 2.7 Data Synthesis and Statistical Analysis

A descriptive analysis was conducted to summarize the study characteristics, baseline demographics, surgical aspects, and postprocedural complications of each included study. Categorical variables were reported as absolute and relative frequencies, while continuous variables were expressed as mean ± standard deviation (SD) when available. These findings were summarized in tables for clarity.

Statistical analyses were performed using the “meta” package in R (version 4.1.2, The R Foundation, 2021). Continuous outcomes were synthesized as mean differences (MD) with 95% confidence intervals (CIs). Pooled estimates were calculated using weighted means (inverse variance method) and reported as mean ± SD.

When individual studies reported medians with ranges or interquartile ranges, mean ± SD values were estimated using the method (Wan et al. 2014). For studies providing MD with CIs or *p* values, SDs were derived using the Cochrane RevMan Calculator (Cochrane Training 2025). If these methods were not feasible, SDs from studies with similar means within the meta‐analysis were used.

Heterogeneity across studies was assessed using Cochran's *Q* test and the *I* ^2^ statistic. Significant heterogeneity was defined as *p* < 0.10 or *I* ^2^ > 25%. For moderate (25 ≤ *I* ^2^ < 50%) or high heterogeneity (*I* ^2^ ≥ 50%), a restricted maximum likelihood random‐effects model was applied. When heterogeneity was low (*I* ^2^ < 25%), a fixed‐effects model was used.

Quantitative data synthesis focused on opioid consumption on postoperative days 1 (POD1: up to 24 h) and 2 (POD2: 24–48 h), pain scores on postoperative days 1 and 3 (POD3: 48–72 h), and hospital length of stay. Intervention groups included patients receiving either LB alone or a combination of LB and PB (LB + PB). Analyses were conducted on the pooled intervention group (LB ± PB), with planned subgroup analyses for LB + PB when feasible.

To address sources of heterogeneity, we combined findings from quality and bias assessments with *I* ^2^ statistics. Sensitivity analyses were conducted by excluding studies contributing most to moderate or high heterogeneity. Additionally, subgroup analyses were performed excluding studies in which the control group involved continuous bupivacaine infusion.

## 3 Results

### 3.1 Study Selection

**FIGURE 1:**
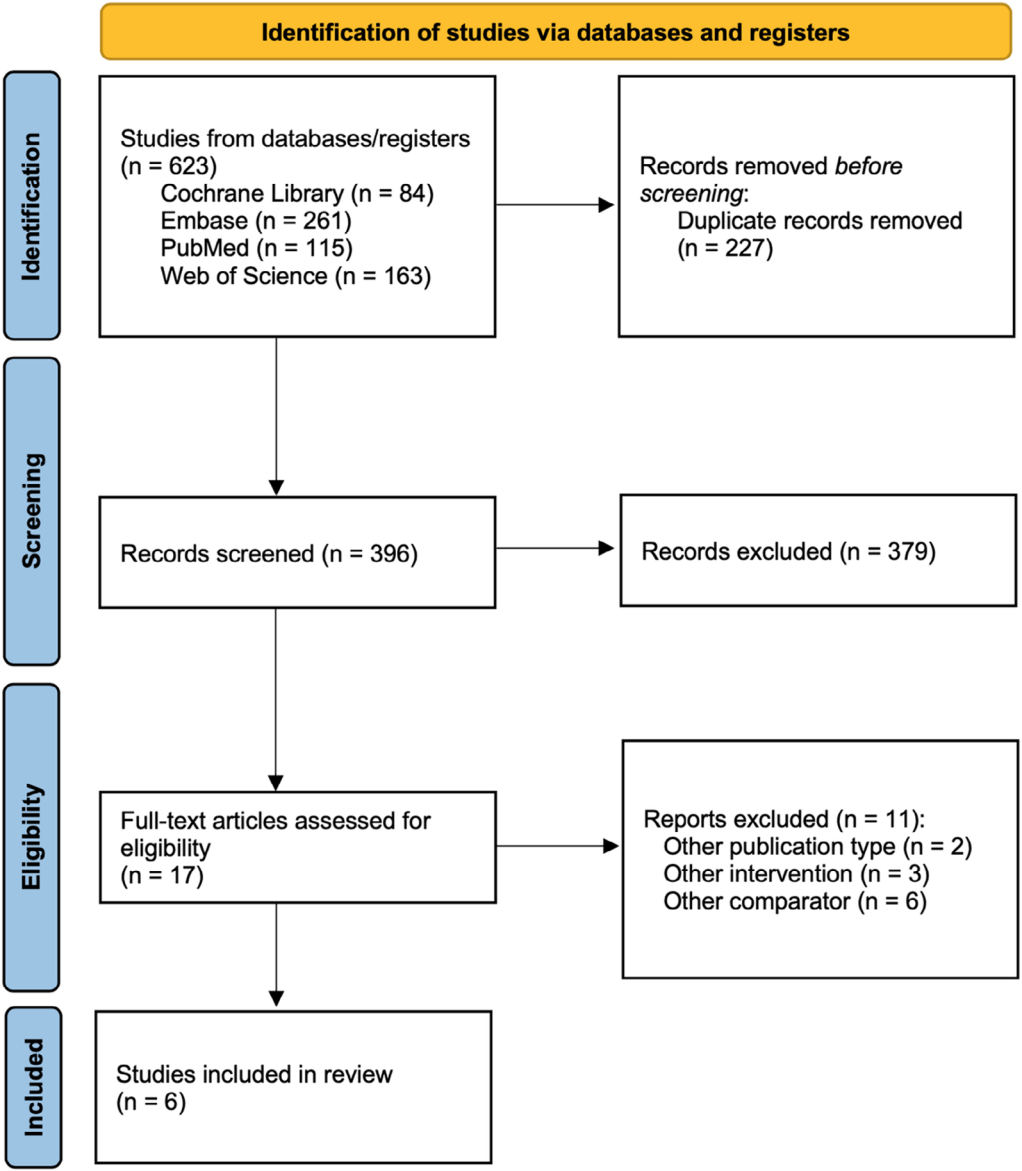
PRISMA flowchart. PRISMA flow diagram of study screening and selection.

A comprehensive search retrieved 84 studies from the Cochrane Library, 261 from Embase, 115 from PubMed, and 163 from Web of Science. After eliminating duplicate records, 396 unique studies remained. A full‐text review was conducted for 17 studies, and following the application of inclusion and exclusion criteria, six studies were deemed eligible for the final meta‐analysis (Rendon et al. 2022; Ha et al. 2019; Nguyen et al. 2024; Park et al. 2024; Jablonka et al. 2017; Gatherwright et al. 2018) (Figure 1).

### 3.2 Characterization of Studies

Among the included studies, four were RCTs and two were retrospective observational studies. All of them were conducted in the USA. Our meta‐analysis included 197 patients in the intervention group and 232 in the control group, all of whom were female and underwent unilateral or bilateral abdominal‐based autologous breast reconstruction (Table 2). A summary of baseline participant characteristics, surgical aspects, and postprocedural complications is available in Tables S2 and S3.

**TABLE 2:** Summary of the main characteristics of the included studies.

Study	Country	Study design	Procedure	Study groups of interest	Intervention(s)	Age ± SD	BMI ± SD	Outcomes available
Gatherwright et al. (2018)	USA	RCT	Unilateral DIEP flap reconstruction	LB TAP block (*n* = 8) versus (2) PB TAP block (*n* = 8) + (3) continuous PB TAP infusion (*n* = 5)	(1) 266 mg LB + 60 mL with 20 mL of 0.25% PB; (2) 2 mg/kg of 0.25% PB; (3) On‐Q pump placed by means of ultrasound guidance in TAP block plane and run at a rate of 0.25% PB at 4 mL/h (0.01 g/h)	(1) 52.1; (2) 53.1; (3) 50	(1) 30; (2) 28; (3) 28	Pain scores on Visual Analogue Scale (POD1 and POD2), length of stay
Ha et al. (2019)	USA	RCT (2016–2018)	Abdominally based autologous breast reconstruction	LB TAP block (*n* = 22) versus PB TAP block (*n* = 22)	266 mg LB versus 75 mg PB	49 ± 9.2 versus 49 ± 10.0	29.1 ± 4.6 versus 28.1 ± 4.5	Pain scores on Numeric Rating Scale (POD1‐3), length of stay
Jablonka et al. (2017)	USA	Retrospective review (2010–2015)	Abdominally based autologous breast reconstruction	LB TAP block (*n* = 40, 62 flaps) versus continuous PB TAP infusion (*n* = 48, 66 flaps)	20 mL of 1.3% LB + 30 mL of 0.25% PB + 80 mL of normal saline versus 30 mL of 0.25% PB injected bilaterally, and then bilateral epidural catheters 0.25% PB (On‐Q pump, 2 mL/h)	50.2 ± 8.5 versus 50.6 ± 8.8	28.0 ± 5.4 versus 26.2 ± 5.0	Opioid consumption (POD1 and POD2), length of stay
Nguyen et al. (2024)	USA	RCT (2019–2020)	Abdominally based autologous breast reconstruction	LB + PB TAP block (*n* = 30) versus PB TAP block (*n* = 30)	266 mg (20 mL) of 1.3% LB + 20 mL of 0.25% versus 20 mL of 0.25% PB	53.0 ± 9.5 versus 52.2 ± 9.8	29.6 ± 5.3 versus 30.2 ± 4.3	Opioid consumption (POD1 and POD2), pain scores on Numeric Pain Scale (POD1‐3), length of stay
Park et al. (2024)	USA	RCT (2021–2022)	DIEP flap breast reconstruction	LB + PB + epinephrine TAP block (*n* = 58) versus PB + epinephrine TAP block (*n* = 59)	30 mL of 0.25% PB, 0.15 mL of 1:1000 epinephrine, and 50 mL of normal saline, and 20 mL of LB (266 mg) versus 30 mL of 0.25% PB, 0.15 mL of 1:1000 epinephrine, and 50 mL of normal saline	51.1 ± 8.7 versus 51.9 ± 10.5	30.0 ± 6.3 versus 31.6 ± 6.7	Opioid consumption (POD1 and POD2), pain scores on Visual Analogue Scale (POD1 and POD2), length of stay
Rendon et al. (2022)	USA	Retrospective cohort (2015–2027)	Autologous breast reconstruction	LB TAP block (*n* = 39) versus continuous PB TAP infusion (*n* = 60)	60 mL of a mixture containing 266 mg of LB + 120 mL saline injected as two equal doses into each TAP plane versus 400 mL of 0.5% PB with an infusion of rate of 4 mL/h	54.9 ± 8.9 versus 50.3 ± 10.4	31.3 ± 5.0 versus 30.1 ± 5.7	Pain scores on Verbal Analog Scale (POD1‐3), length of stay

Abbreviations: DIEP, Deep Inferior Epigastric Perforator; LB, liposomal bupivacaine; PB, plain bupivacaine; POD, postoperative day; RCT, randomized controlled trial; TAP, Transversus Abdominis Plane.

The intervention group received one of the following: (1) a LB TAP block, (2) a combination of LB and PB TAP block, or (3) a mix of LB, PB, and epinephrine TAP block. The control group received (1) a PB TAP block, (2) a combination of PB and epinephrine TAP block, or (3) a continuous PB TAP block (Table 2). Further details in different perioperative protocols used in each study can be found in Table S4.

### 3.3 Quality and Bias Assessment

Among the RCTs evaluated using the ROB 2 tool, three studies demonstrated a low risk of bias across all assessed domains (Nguyen et al. 2024; Park et al. 2024; Gatherwright et al. 2018). One study presented some concerns in the measurement of the outcome, which affected its overall judgment (Ha et al. 2019) (Figure S1). Non‐randomized studies assessed with the ROBINS‐I tool both exhibited a moderate overall risk of bias. Jablonka et al. had a moderate risk primarily due to concerns related to confounding, outcome measurement, and deviations from intended interventions (Jablonka et al. 2017). Similarly, Rendon et al. (2022) had a moderate overall risk, with concerns in confounding, participant selection, and outcome measurement (Figure S2).

The funnel plot analysis appears asymmetrical, with studies distributed unevenly around the mean effect size. Notably, (Jablonka et al. 2017) deviate substantially from the center, suggesting potential publication bias or heterogeneity in study (Figure S3).

### 3.4 Opioid Consumption

**FIGURE 2:**
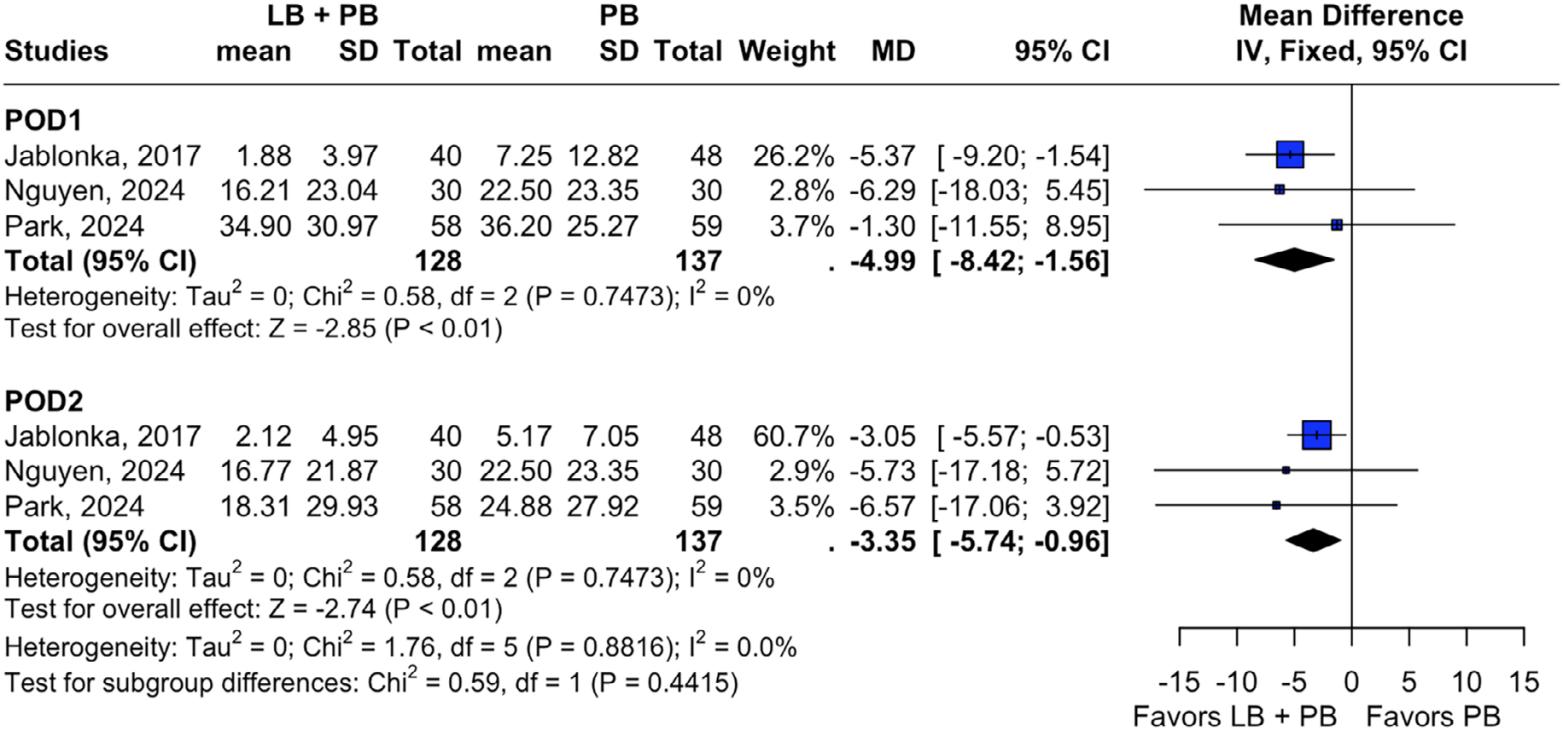
Forest plot showing opioid consumption outcomes in oral morphine equivalents. Opioid consumption on postoperative days 1 and 2 comparing liposomal bupivacaine plus plain bupivacaine versus plain bupivacaine alone. CI, confidence interval; IV, inverse variance; LB, liposomal bupivacaine; MD, mean difference; PB, plain bupivacaine; POD, postoperative day; SD, standard deviation.

This analysis involved three studies including 128 patients in the LB + PB group and 137 in the PB group and compared opioid consumption between them on POD1 and POD2 in oral morphine equivalents (OME) (Nguyen et al. 2024; Park et al. 2024; Jablonka et al. 2017). On POD1, opioid use was significantly lower in the LB + PB group, with a mean difference of −4.99 OME (95% CI [−8.42; −1.56], *p* < 0.01, *I* ^2^ = 0%). On POD2, the reduction in opioid consumption remained significant (MD = −3.35, 95% CI [−5.74; −0.96], *p* < 0.01, *I* ^2^ = 0%) (Figure 2). (Jablonka et al. 2017) contributed the most to the pooled effect on both days, with a mean difference of −5.37 (95% CI [−9.20; −1.54]) on POD1 and −3.05 (95% CI [−5.57; −0.53]) on day 2 (Jablonka et al. 2017). The combination of LB + PB significantly reduced opioid requirements over the first 48 h postoperatively compared to PB alone.

### 3.5 Pain Score Analysis

**FIGURE 3:**
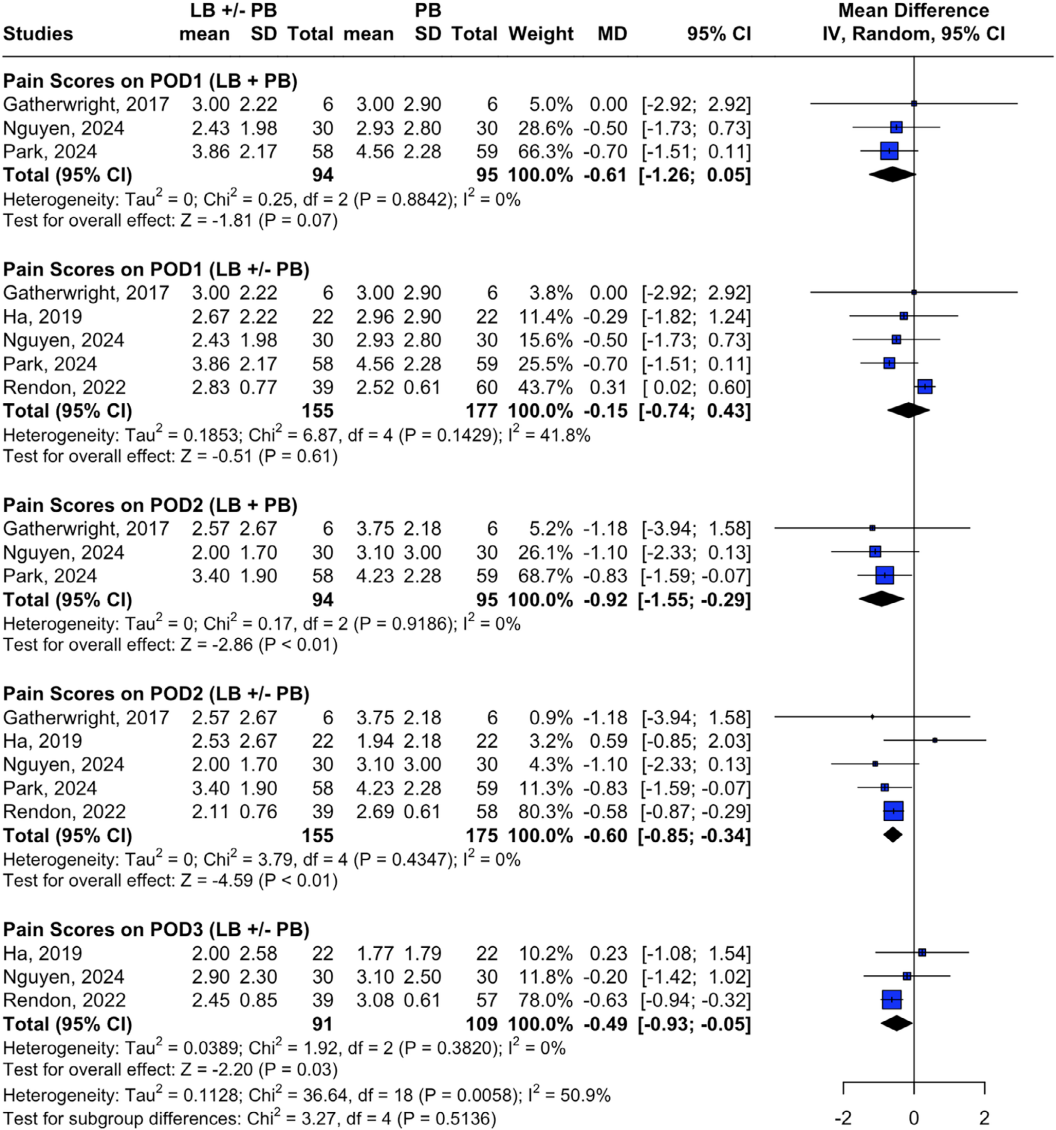
Forest plot showing pain score outcomes. Pain scores on postoperative day 1 and postoperative day 3 among liposomal bupivacaine, with or without plain bupivacaine, versus plain bupivacaine alone. *(Ha et al. 2019 and Rendon et al. 2020) compared liposomal bupivacaine alone versus plain bupivacaine alone. CI, confidence interval; IV, inverse variance; LB, liposomal bupivacaine; MD, mean difference; PB, plain bupivacaine; POD, postoperative day; SD, standard deviation.

Postoperative pain scores over the first 72 h evaluated the impact of LB with or without PB compared to PB alone. Five studies reported pain scores, all of them using patient‐reported scales ranging from 0 to 10 (Rendon et al. 2022; Ha et al. 2019; Nguyen et al. 2024; Park et al. 2024; Gatherwright et al. 2018). On POD1, there was no significant difference in pain scores between the intervention and control groups. The pooled MD for LB + PB versus PB was −0.61 (95% CI [−1.26; 0.05], *p* = 0.07, *I* ^2^ = 0%), while for LB with or without PB versus PB, the MD was −0.15 (95% CI: [−0.74; 0.43], *p* = 0.61, *I* ^2^ = 41.8%) (Figure 3). A sensitivity analysis, excluding Rendon et al. (2022) from the LB with or without PB versus PB comparison, reduced heterogeneity to zero while maintaining the overall conclusion (MD = −0.56, 95% CI [−1.16, 0.05], *p* = 0.07, *I* ^2^ = 0) (Figure S4).

On POD2, a statistically significant reduction in pain scores was observed in the intervention group compared to the control. The MD for LB + PB versus PB was −0.92 (95% CI [−1.55; −0.29], *p* < 0.01, *I* ^2^ = 0%), and for LB with or without PB versus PB, the MD was −0.60 (95% CI [0.85; −0.34], *p* < 0.01, *I* ^2^ = 0%). By POD3, pain scores continued to be significantly lower in the intervention group. The pooled mean difference was −0.49 (95% CI: [−0.93; −0.05], *p* = 0.03, *I* ^2^ = 0%) (Figure 3).

### 3.6 Length of Stay

**FIGURE 4:**
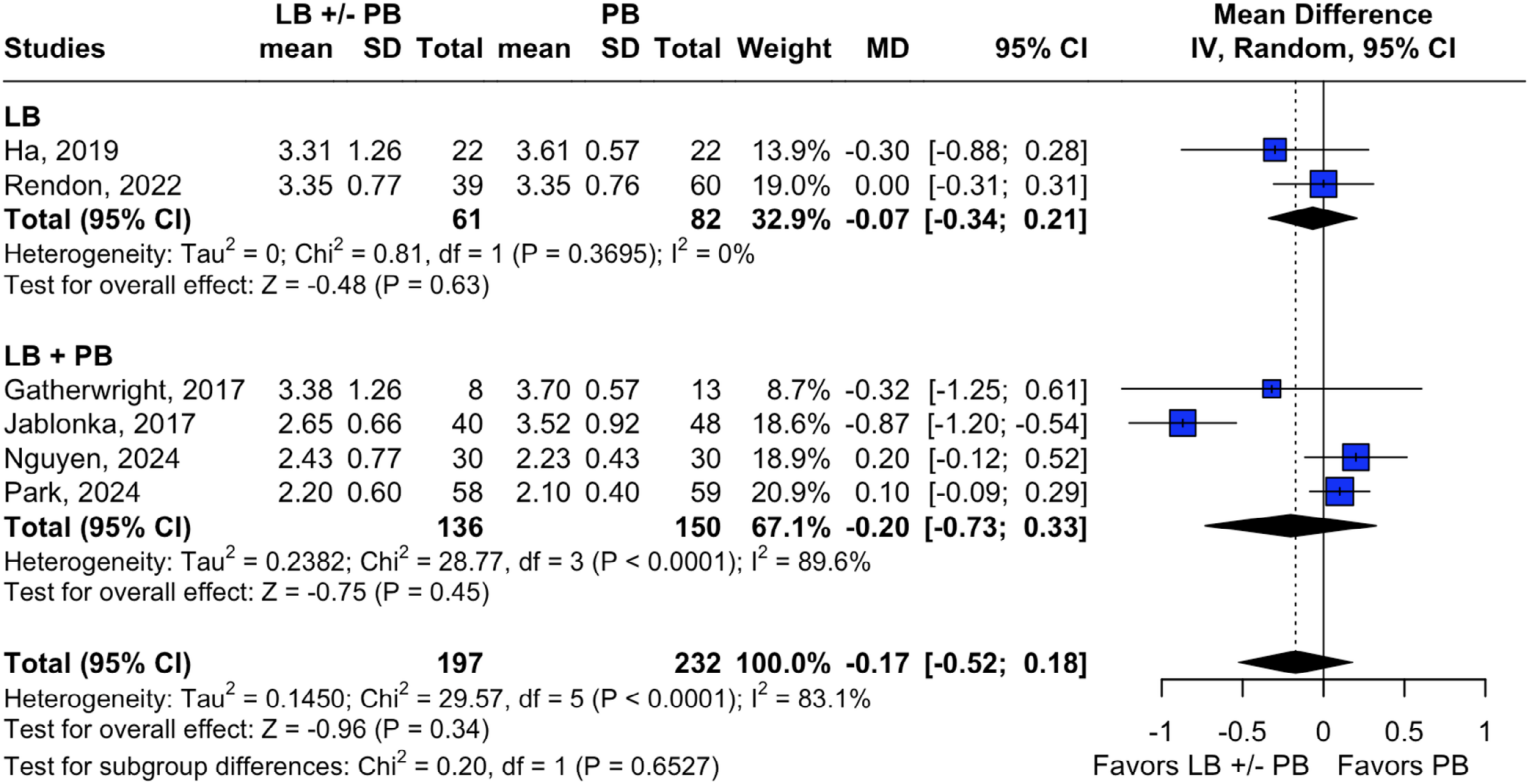
Forest plot illustrating the assessment of length of stay outcomes in days. Length of stay among liposomal bupivacaine, with or without plain bupivacaine, versus plain bupivacaine alone. *Gatherwright et al. (2018) included a subgroup of patients receiving continuous bupivacaine TAP infusion in the control group for this analysis. Jablonka et al. (2017) administered continuous bupivacaine TAP infusion to all patients in the control group. CI, confidence interval; IV, inverse variance; LB, liposomal bupivacaine; MD, mean difference; PB, plain bupivacaine; POD, postoperative day; SD, standard deviation.

There was no significant reduction in the length of hospital stay in the intervention groups compared to PB alone (Rendon et al. 2022; Ha et al. 2019; Nguyen et al. 2024; Park et al. 2024; Jablonka et al. 2017; Gatherwright et al. 2018). For LB alone versus PB, pooled MD was −0.07 (95% CI [−0.34; 0.21], *p* = 0.63, *I* ^2^ = 0%), while for LB + PB versus PB, MD was −0.20 (95% CI [−0.73; 0.33], *p* = 0.45, *I* ^2^ = 89.6%). When pooling all studies together, the overall MD was −0.17 (95% CI [−0.52; 0.18], *p* = 0.34, *I* ^2^ = 83.1%) (Figure 4).

Jablonka et al. (2017) was the only study to report a significant reduction in length of stay (MD = −0.87, 95% CI [−1.20, −0.54]). This study contributed substantially to the high heterogeneity observed in the pooled analysis. When it was removed, heterogeneity dropped to zero; however, there remained no significant difference in length of stay between the intervention and control groups (MD = 0.07, 95% CI [−0.07, 0.20], *p* = 0.52, *I* ^2^ = 0) (Jablonka et al. 2017) (Figure S5). Similar findings were observed when patients receiving continuous PB TAP infusion in Gatherwright et al. and Jablonka et al. were excluded (MD = 0.07, 95% CI [−0.07, 0.21], *p* = 0.57, *I* ^2^ = 0) (Jablonka et al. 2017; Gatherwright et al. 2018) (Figure S6).

## 4 Discussion

In this systematic review and meta‐analysis, we compared the postoperative outcomes of women who underwent TAP block with LB versus the standard (PB) following autologous breast reconstruction. The principal findings in the group of patients who received LB, with or without the addition of PB, were as follows: (1) reduced opioid consumption on POD1 and POD2; (2) no significant difference in pain scores on POD1; (3) lower pain levels on POD2 and POD3; and (4) no significant difference in hospital length of stay.

Opioid use remains a significant public health concern in the United States, particularly given its association with a substantial increase in mortality and adverse events over the past several decades (Little et al. 2019). While opioids are widely utilized for postoperative pain management, they are accompanied by numerous side effects, including nausea, vomiting, respiratory depression, pruritus, opioid‐induced hyperalgesia, prolonged ileus, sedation, dizziness, physical dependence, increased hospital length of stay, and escalated healthcare costs (Little et al. 2019; Chahar and Cummings 2012). Thus, strategies that mitigate opioid consumption in the postoperative period offer considerable potential benefits for patients in both the short and long term.

In our pooled analysis, the combination of LB and PB was associated with a statistically significant reduction in opioid consumption on both POD1 and POD2. However, this result was largely driven by the study by (Jablonka et al. 2017), which, despite its methodological rigor, was not a RCT. The RCTs by (Nguyen et al. 2024 and Park et al. 2024) did not individually reach statistical significance but showed consistent trends favoring the LB + PB group. This consistency, though not definitive, suggests a potential opioid‐sparing effect that may have been underestimated due to limited sample sizes and study power. These findings highlight the need for larger, well‐designed RCTs to provide more robust and clinically meaningful evidence regarding the impact of LB on postoperative opioid consumption.

Nonetheless, the magnitude of the reduction—4.99 and 3.35 mg OME on POD1 and POD2, respectively—should be interpreted with caution. Across studies, average total opioid consumption on POD1 ranged from 1.88 to 36.20 mg OME, with a maximum average of 24.88 mg on POD2. The reductions in our meta‐analysis represent roughly 15% of the highest reported averages, suggesting a potentially relevant numerical difference. However, its clinical significance remains uncertain. These amounts are less than a standard dose of oral oxycodone and may be too small to meaningfully impact functional recovery, opioid‐related side effects, or long‐term outcomes (Dowell et al. 2016). Notably, (Laigaard et al. 2021) proposed a minimal clinically important difference of 40 mg OME over 24 h in the context of total hip and knee arthroplasty—substantially greater than the reductions observed here. While derived from a different surgical population, this threshold highlights that the modest opioid reductions in our study likely fall below what is necessary to achieve perceptible clinical benefits. Therefore, although our findings support the analgesic potential of LB, larger RCTs are needed to determine whether these reductions translate into meaningful improvements in postoperative recovery and opioid stewardship in reconstructive surgery.

LB consists of bupivacaine encapsulated in multivesicular liposomes, which prolong the duration of local anesthesia and extend the release time of the liposome (BUPI), thereby delaying the peak plasma concentration when compared to plain bupivacaine (Chahar and Cummings 2012; Manna et al. 2019; Hu et al. 2013; Malik et al. 2017). Its administration results in systemic plasma levels of bupivacaine that can persist for up to 96 h following local infiltration and up to 120 h after interscalene brachial plexus block (Pacira BioSciences Inc. 2018; Cohen 2012).

Therefore, the extended anesthetic effect of LB likely accounts for the observation in our meta‐analysis that, while LB—either alone or in combination with PB—did not provide significant pain relief in comparison to PB alone in the immediate postoperative period (POD1), its benefits became more pronounced on the second (POD2) and third postoperative days (POD3). However, although these delayed improvements in pain scores were statistically significant, their clinical relevance also remains uncertain and warrants cautious interpretation.

Gallagher et al. proposed that a minimum reduction of 1.6 points on the visual analog scale (VAS) is necessary to achieve clinically meaningful relief in acute abdominal pain (Gallagher et al. 2002). Similarly, (Myles et al. 2017) identified a minimal clinically important difference of 0.99 points on the VAS for postoperative pain. In our study, the observed pain score reductions ranged from 0.49 to 0.92 points, which, although statistically significant, falls below these established thresholds for clinical significance. These findings suggest that while LB may provide some analgesic benefit, its impact on patient‐perceived pain improvement during this timeframe may be modest.

Effective pain control is a critical component of postoperative care in abdominal surgeries, as inadequate analgesia has been associated with adverse outcomes including impaired respiratory mechanics, increased psychological distress, and prolonged recovery in various clinical contexts (Little et al. 2019; Oliveira et al. 2012). Although the reductions in pain scores observed in our study were modest, they may contribute to incremental improvements in patient comfort and recovery, underscoring the need for ongoing research into optimizing analgesic strategies.

While LB is associated with higher initial financial costs, it may lead to cost savings in the long term by reducing the incidence of postoperative complications related to inadequate pain control and opioid use, promoting earlier functional recovery, and preventing the onset of chronic pain (Little et al. 2019; Salibian et al. 2018). In our meta‐analysis, we found no significant difference in hospital length of stay between patients who received LB and those who received plain bupivacaine. This finding may be influenced by factors unrelated to analgesic efficacy, including heterogeneity in perioperative protocols, institutional discharge policies, and insurance‐related constraints. These variables—particularly in the United States—can standardize early discharge regardless of patient‐reported pain scores or functional readiness (Ljungqvist et al. 2017; Wick et al. 2017). Therefore, although hospital length of stay remains a commonly reported and potentially informative variable, it may have limited value in reflecting the true impact of pain management strategies. Future research might benefit from incorporating alternative endpoints more directly related to functional recovery, such as time to ambulation, gastrointestinal recovery, or validated patient‐centered recovery measures.

Our systematic review and meta‐analysis has several limitations. Despite the overall trends observed, interpretation of the findings requires caution due to heterogeneity in ERAS protocols (Kaoutzanis et al. 2018; Odom et al. 2017), outcome reporting, and anesthetic techniques. Perioperative analgesic strategies varied across studies, including differences in the use of systemic medications and additional regional blocks. A notable example is the use of catheter‐based PB infusion, which differs from the single‐shot TAP block technique (Jablonka et al. 2017; Gatherwright et al. 2018). While continuous infusion may prolong analgesia, it can also restrict mobility and delay discharge.

Another important consideration is the inconsistency in perioperative analgesic protocols. While some studies followed structured ERAS pathways with scheduled non‐opioid medications, others lacked detailed reporting on adjunctive analgesia, limiting interpretability (Rendon et al. 2022; Gatherwright et al. 2018). Additionally, certain protocols included other regional blocks, such as paravertebral and erector spinae plane blocks, which may have influenced outcomes independently of the TAP block (Ha et al. 2019; Park et al. 2024). Reporting of opioid consumption also varied, with some studies presenting data by perioperative phase (Rendon et al. 2022; Ha et al. 2019) and others using cumulative hourly intervals (Nguyen et al. 2024; Park et al. 2024; Jablonka et al. 2017), limiting a more robust pooled analysis of opioid use. Despite these differences, all studies reporting pain scores were eligible for inclusion in the pooled analysis of postoperative pain, which consistently favored the use of LB (Rendon et al. 2022; Ha et al. 2019; Nguyen et al. 2024; Park et al. 2024; Gatherwright et al. 2018). These findings highlight the need for greater standardization in perioperative protocols and outcome reporting.

Furthermore, postoperative complications were inconsistently reported across studies, with different studies evaluating different outcomes. This heterogeneity precluded a quantitative synthesis of complications. However, among those reported in more than one study—graft loss, hematoma, bleeding, return to the operation room, and readmission—incidence was comparable between groups. This finding is consistent with a previous systematic review, suggesting that LB does not pose additional risks for these outcomes (Vyas et al. 2016). Finally, none of the studies evaluated hospitalization costs, leaving the cost‐effectiveness of this therapeutic approach an important area for future investigation.

## 5 Conclusion

The local infiltration of LB, either alone or in combination with PB, into the TAP block during autologous breast reconstruction, when compared to PB, was associated with reduced opioid consumption during the first 48 h postoperatively and modest improvements in pain scores on POD 2 and POD 3, without any significant difference in hospital length of stay. While these findings suggest potential analgesic benefits, the magnitude of effect may fall below clinically significant thresholds. Further large‐scale, RCTs are warranted to better define the role of LB in optimizing postoperative recovery and to clarify its impact on patient‐centered outcomes and healthcare resource utilization.

## Disclosure

The authors have nothing to report.

## Conflicts of Interest

The authors declare no conflicts of interest.

## Supporting information


**Figure S1:** micr70126‐sup‐0001‐FigureS1.png.

**Figure S2:** micr70126‐sup‐0002‐FigureS2.png.

**Figure S3:** micr70126‐sup‐0003‐FigureS3.png.

**Figure S4:** micr70126‐sup‐0004‐FigureS4.png.

**Figure S5:** micr70126‐sup‐0005‐FigureS5.png.

**Figure S6:** micr70126‐sup‐0006‐FigureS6.png.

**Table S1:** micr70126‐sup‐0007‐TableS1.docx.

**Table S2:** micr70126‐sup‐0008‐TableS2.docx.

**Table S3:** micr70126‐sup‐0009‐TableS3.docx.

**Table S4:** micr70126‐sup‐0010‐TableS4.docx.

## Data Availability

All data generated or analyzed during this review is available to the corresponding author upon request.

## References

[micr70126-bib-0001] Afonso, A. , S. Oskar , K. S. Tan , et al. 2017. “Is Enhanced Recovery the New Standard of Care in Microsurgical Breast Reconstruction?” Plastic and Reconstructive Surgery 139, no. 4: 1053–1061. 10.1097/PRS.0000000000003240. 28092334 PMC5640259

[micr70126-bib-0002] Afonso, A. M. , M. I. Newman , N. Seeley , et al. 2017. “Multimodal Analgesia in Breast Surgical Procedures: Technical and Pharmacological Considerations for Liposomal Bupivacaine Use.” Plastic and Reconstructive Surgery. Global Open 5, no. 11: e1480. 10.1097/GOX.0000000000001480. 29062649 PMC5640354

[micr70126-bib-0003] American Society of Plastic Surgeons . 2023. 2023 Plastic Surgery Statistics Report. https://www.plasticsurgery.org/documents/news/statistics/2023/plastic‐surgery‐statistics‐report‐2023.pdf.

[micr70126-bib-0004] Batdorf, N. J. , V. Lemaine , J. K. Lovely , et al. 2015. “Enhanced Recovery After Surgery in Microvascular Breast Reconstruction.” Journal of Plastic, Reconstructive & Aesthetic Surgery 68, no. 3: 395–402. 10.1016/j.bjps.2014.11.020. 25488326

[micr70126-bib-0005] Chahar, P. , and K. C. Cummings . 2012. “Liposomal Bupivacaine: A Review of a New Bupivacaine Formulation.” Journal of Pain Research 5: 257–264. 10.2147/JPR.S27894. 23049275 PMC3442744

[micr70126-bib-0006] Cochrane Training . 2025. RevMan Calculator. https://training.cochrane.org/resource/revman‐calculator.

[micr70126-bib-0007] Cohen, S. M. 2012. “Extended Pain Relief Trial Utilizing Infiltration of Exparel, a Long‐Acting Multivesicular Liposome Formulation of Bupivacaine: A Phase IV Health Economic Trial in Adult Patients Undergoing Open Colectomy.” Journal of Pain Research 5: 567–572. 10.2147/JPR.S38621. 23204866 PMC3508659

[micr70126-bib-0008] Dowell, D. , T. M. Haegerich , and R. Chou . 2016. “CDC Guideline for Prescribing Opioids for Chronic Pain—United States, 2016.” MMWR Recommendations and Reports 65, no. 1: 1–49. 10.15585/mmwr.rr6501e1. 26987082

[micr70126-bib-0009] Gallagher, E. J. , P. E. Bijur , C. Latimer , and W. Silver . 2002. “Reliability and Validity of a Visual Analog Scale for Acute Abdominal Pain in the ED.” American Journal of Emergency Medicine 20, no. 4: 287–290. 10.1053/ajem.2002.33778. 12098173

[micr70126-bib-0010] Gatherwright, J. , R. W. Knackstedt , A. M. Ghaznavi , et al. 2018. “Prospective, Randomized, Controlled Comparison of Bupivacaine Versus Liposomal Bupivacaine for Pain Management After Unilateral Delayed Deep Inferior Epigastric Perforator Free Flap Reconstruction.” Plastic and Reconstructive Surgery 141, no. 6: 1327–1336. 10.1097/PRS.0000000000004360. 29750760

[micr70126-bib-0011] Ha, A. Y. , G. Keane , R. Parikh , et al. 2019. “The Analgesic Effects of Liposomal Bupivacaine Versus Bupivacaine Hydrochloride Administered as a Transversus Abdominis Plane Block After Abdominally Based Autologous Microvascular Breast Reconstruction: A Prospective, Single‐Blind, Randomized, Controlled Trial.” Plastic and Reconstructive Surgery 144, no. 1: 35–46. 10.1097/PRS.0000000000005698. 31246796

[micr70126-bib-0012] Higgins, J. P. T. , J. Thomas , J. Chandler , et al. 2022. Cochrane Handbook for Systematic Reviews of Interventions. Cochrane.

[micr70126-bib-0013] Hu, D. , E. Onel , N. Singla , W. G. Kramer , and A. Hadzic . 2013. “Pharmacokinetic Profile of Liposome Bupivacaine Injection Following a Single Administration at the Surgical Site.” Clinical Drug Investigation 33, no. 2: 109–115. 10.1007/s40261-012-0043-z. 23229686

[micr70126-bib-0014] Jablonka, E. M. , A. M. Lamelas , J. N. Kim , et al. 2017. “Transversus Abdominis Plane Blocks With Single‐Dose Liposomal Bupivacaine in Conjunction With a Nonnarcotic Pain Regimen Help Reduce Length of Stay Following Abdominally Based Microsurgical Breast Reconstruction.” Plastic and Reconstructive Surgery 140, no. 2: 240–250. 10.1097/PRS.0000000000003508. 28746269 PMC9074889

[micr70126-bib-0015] Jüni, P. , Y. Loke , T. Pigott , et al. 2016. “Risk of Bias in Non‐Randomized Studies of Interventions (ROBINS‐I): Detailed Guidance.” BMJ 355: i4919. 27733354 10.1136/bmj.i4919 PMC5062054

[micr70126-bib-0016] Kaoutzanis, C. , N. Ganesh Kumar , D. O'Neill , et al. 2018. “Enhanced Recovery Pathway in Microvascular Autologous Tissue‐Based Breast Reconstruction: Should It Become the Standard of Care?” Plastic and Reconstructive Surgery 141, no. 4: 841–851. 10.1097/PRS.0000000000004146. 29465485 PMC5876075

[micr70126-bib-0017] Kehlet, H. 1997. “Multimodal Approach to Control Postoperative Pathophysiology and Rehabilitation.” British Journal of Anaesthesia 78, no. 5: 606–617. 10.1093/bja/78.5.606. 9175983

[micr70126-bib-0018] Knackstedt, R. , J. D. Oliver , and J. Gatherwright . 2020. “Optimizing Postoperative Pain Control in Autologous Breast Reconstruction: A Systematic Review.” Journal of Reconstructive Microsurgery 36, no. 7: 480–485. 10.1055/s-0040-1708834. 32289845

[micr70126-bib-0019] Knackstedt, R. W. , J. H. Lin , and S. Kakoty . 2024. “Liposomal Bupivacaine Analgesia in Deep Inferior Epigastric Perforator Flap Breast Reconstruction: A Retrospective Cohort Study.” Plastic and Reconstructive Surgery. Global Open 12: e5874. 10.1097/GOX.0000000000005874. 38855138 PMC11161287

[micr70126-bib-0020] Laigaard, J. , C. Pedersen , T. N. Rønsbo , O. Mathiesen , and A. P. H. Karlsen . 2021. “Minimal Clinically Important Differences in Randomised Clinical Trials on Pain Management After Total Hip and Knee Arthroplasty: A Systematic Review.” British Journal of Anaesthesia 126, no. 5: 1029–1037. 10.1016/j.bja.2021.01.021. 33678402

[micr70126-bib-0021] Little, A. , K. Brower , D. Keller , B. Ramshaw , and J. E. Janis . 2019. “A Cost‐Minimization Analysis Evaluating the Use of Liposomal Bupivacaine in Reconstructive Plastic Surgery Procedures.” Plastic and Reconstructive Surgery 143, no. 4: 1269–1274. 10.1097/PRS.0000000000005435. 30730499

[micr70126-bib-0022] Ljungqvist, O. , M. Scott , and K. C. Fearon . 2017. “Enhanced Recovery After Surgery: A Review.” JAMA Surgery 152, no. 3: 292–298. 10.1001/jamasurg.2016.4952. 28097305

[micr70126-bib-0023] Malik, O. , A. D. Kaye , A. Kaye , K. Belani , and R. D. Urman . 2017. “Emerging Roles of Liposomal Bupivacaine in Anesthesia Practice.” Journal of Anaesthesiology Clinical Pharmacology 33, no. 2: 151–156. 10.4103/joacp.JOACP_375_15. 28781438 PMC5520585

[micr70126-bib-0024] Manna, S. , Y. Wu , Y. Wang , et al. 2019. “Probing the Mechanism of Bupivacaine Drug Release From Multivesicular Liposomes.” Journal of Controlled Release 294: 279–287. 10.1016/j.jconrel.2018.12.029. 30576748

[micr70126-bib-0025] Myles, P. S. , D. B. Myles , W. Galagher , et al. 2017. “Measuring Acute Postoperative Pain Using the Visual Analog Scale: The Minimal Clinically Important Difference and Patient Acceptable Symptom State.” British Journal of Anaesthesia 118, no. 3: 424–429. 10.1093/bja/aew466. 28186223

[micr70126-bib-0026] Nguyen, L. , G. E. Glassman , A. Afshari , et al. 2024. “Randomized Controlled Trial Comparing Liposomal to Plain Bupivacaine in the Transversus Abdominis Plane for DIEP Flap Breast Reconstruction.” Plastic and Reconstructive Surgery 153, no. 3: 543–555. 10.1097/PRS.0000000000010710. 37220228

[micr70126-bib-0027] Odom, E. B. , N. Mehta , R. P. Parikh , R. Guffey , and T. M. Myckatyn . 2017. “Paravertebral Blocks Reduce Narcotic Use Without Affecting Perfusion in Patients Undergoing Autologous Breast Reconstruction.” Annals of Surgical Oncology 24: 3180–3187. 28718036 10.1245/s10434-017-6007-z PMC6136427

[micr70126-bib-0028] Oliveira, R. M. , S. B. Tenório , P. P. Tanaka , and D. Precoma . 2012. “Control of Pain Through Epidural Block and Incidence of Cardiac Dysrhythmias in Postoperative Period of Thoracic and Major Abdominal Surgical Procedures: A Comparative Study.” Revista Brasileira de Anestesiologia 62, no. 1: 10–18. 22248761 10.1016/S0034-7094(12)70098-3

[micr70126-bib-0029] Pacira BioSciences Inc . 2018. EXPAREL (Bupivacaine Liposome Injectable Suspension). www.accessdata.fda.gov/drugsatfda_docs/label/2018/022496s9lbl.pdf.

[micr70126-bib-0030] Page, M. J. , J. E. McKenzie , P. M. Bossuyt , et al. 2021. “The PRISMA 2020 Statement: An Updated Guideline for Reporting Systematic Reviews.” BMJ 372: n71. 33782057 10.1136/bmj.n71 PMC8005924

[micr70126-bib-0031] Park, R. H. , J. Chou , R. G. DeVito , et al. 2024. “Effectiveness of Liposomal Bupivacaine Transversus Abdominis Plane Block in DIEP Flap Breast Reconstruction: A Randomized Controlled Trial.” Plastic and Reconstructive Surgery 154, no. 4S: 52S–60S. 10.1097/PRS.0000000000011326. 38315156

[micr70126-bib-0032] Rendon, J. L. , J. Borrell‐Vega , J. P. C. Reyes , et al. 2022. “Evaluating the Efficacy of Two Regional Pain Management Modalities in Autologous Breast Reconstruction.” Plastic and Reconstructive Surgery. Global Open 10: e4010. 10.1097/GOX.0000000000004010. 35070591 PMC8769083

[micr70126-bib-0033] Rendon, J. L. , T. Hodson , R. J. Skoracki , et al. 2020. “Enhanced Recovery After Surgery Protocols Decrease Outpatient Opioid Use in Patients Undergoing Abdominally Based Microsurgical Breast Reconstruction.” Plastic and Reconstructive Surgery 145, no. 3: 645–651. 10.1097/PRS.0000000000006585. 32097300

[micr70126-bib-0034] Salibian, A. A. , J. D. Frey , V. D. Thanik , N. S. Karp , and M. Choi . 2018. “Transversus Abdominis Plane Blocks in Microsurgical Breast Reconstruction: Analysis of Pain, Narcotic Consumption, Length of Stay, and Cost.” Plastic and Reconstructive Surgery 142, no. 3: 252e–263e. 10.1097/PRS.0000000000004632. 29879000

[micr70126-bib-0035] Sebai, M. E. , C. Siotos , R. Payne , et al. 2019. “Enhanced Recovery After Surgery Pathway for Microsurgical Breast Reconstruction: A Systematic Review and Meta‐Analysis.” Plastic and Reconstructive Surgery 143, no. 3: 655–666. 10.1097/PRS.0000000000005411. 30589825

[micr70126-bib-0036] Sterne, J. A. C. , J. Savović , M. J. Page , et al. 2019. “RoB 2: A Revised Tool for Assessing Risk of Bias in Randomised Trials.” BMJ 366: l4898. 10.1136/bmj.l4898. 31462531

[micr70126-bib-0037] Vyas, K. S. , S. Rajendran , S. D. Morrison , et al. 2016. “Systematic Review of Liposomal Bupivacaine (Exparel) for Postoperative Analgesia.” Plastic and Reconstructive Surgery 138, no. 4: 748e–756e. 10.1097/PRS.0000000000002547. 27673545

[micr70126-bib-0038] Wan, X. , W. Wang , J. Liu , and T. Tong . 2014. “Estimating the Sample Mean and Standard Deviation From the Sample Size, Median, Range and/or Interquartile Range.” BMC Medical Research Methodology 14: 135. 25524443 10.1186/1471-2288-14-135 PMC4383202

[micr70126-bib-0039] Wick, E. C. , M. C. Grant , and C. L. Wu . 2017. “Postoperative Multimodal Analgesia Pain Management With Nonopioid Analgesics and Techniques: A Review.” JAMA Surgery 152, no. 7: 691–697. 10.1001/jamasurg.2017.0898. 28564673

