# Supplementary figures and images for: Liposomal Bupivacaine in Transversus Abdominis Plane Block for Postoperative Pain Control After Autologous Breast Reconstruction: A Systematic Review and Meta‐Analysis
Source: Microsurgery. 2025 Oct 3;45(7):e70126. doi: 10.1002/micr.70126 (PMC12493009; doi:10.1002/micr.70126)

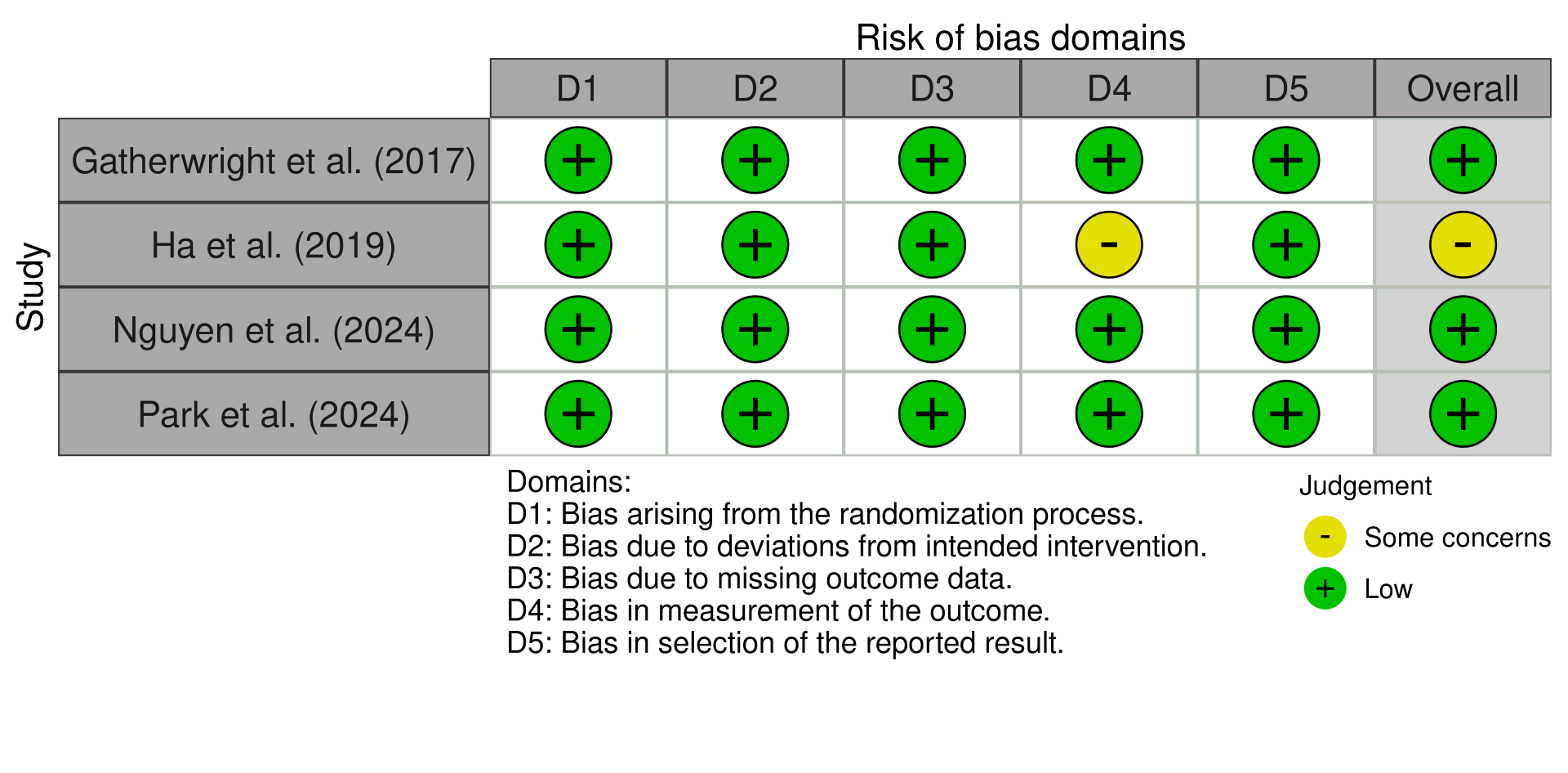

Supplement: Supplementary file 1 — Figure S1: micr70126‐sup‐0001‐FigureS1.png. [file MICR-45-e70126-s005.png]

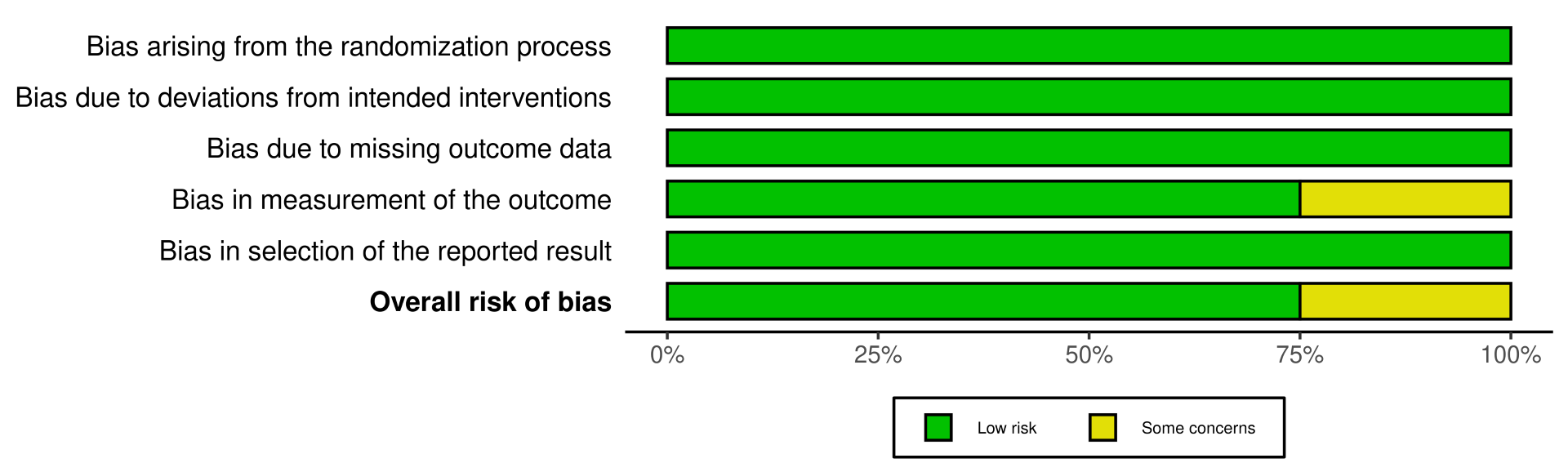

Supplement: Supplementary file 2 — Figure S2: micr70126‐sup‐0002‐FigureS2.png. [file MICR-45-e70126-s006.png]

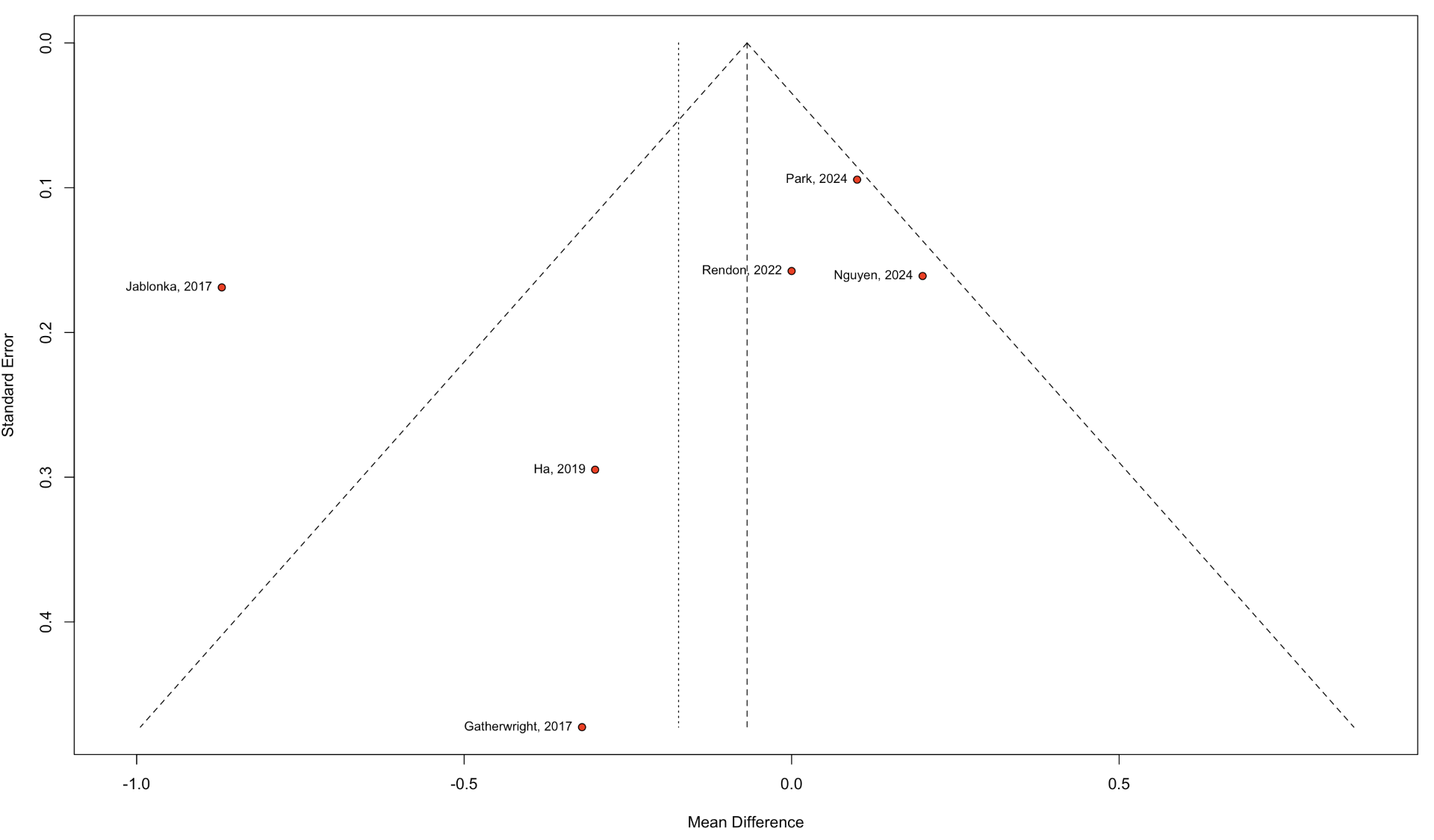

Supplement: Supplementary file 3 — Figure S3: micr70126‐sup‐0003‐FigureS3.png. [file MICR-45-e70126-s001.png]

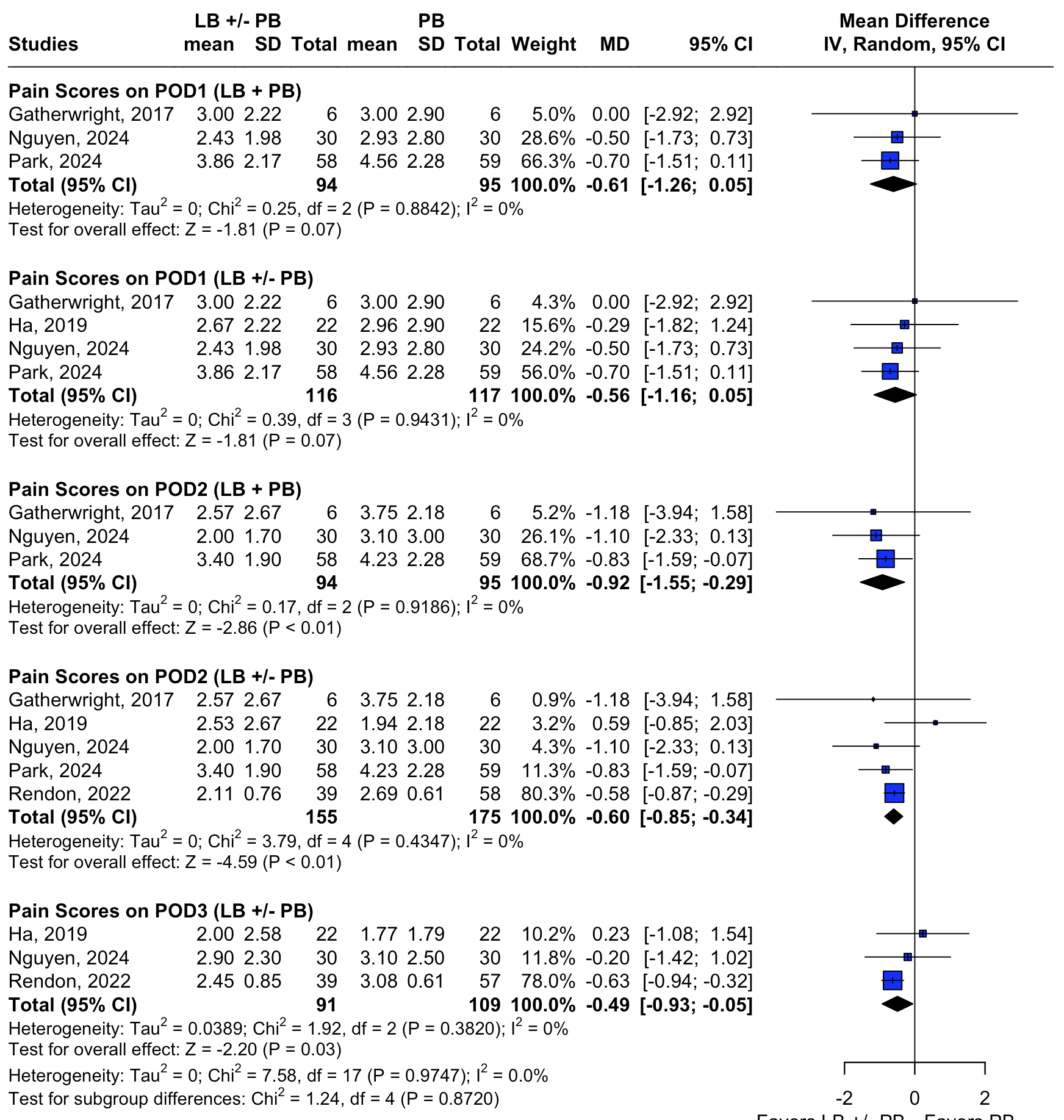

Supplement: Supplementary file 4 — Figure S4: micr70126‐sup‐0004‐FigureS4.png. [file MICR-45-e70126-s009.png]

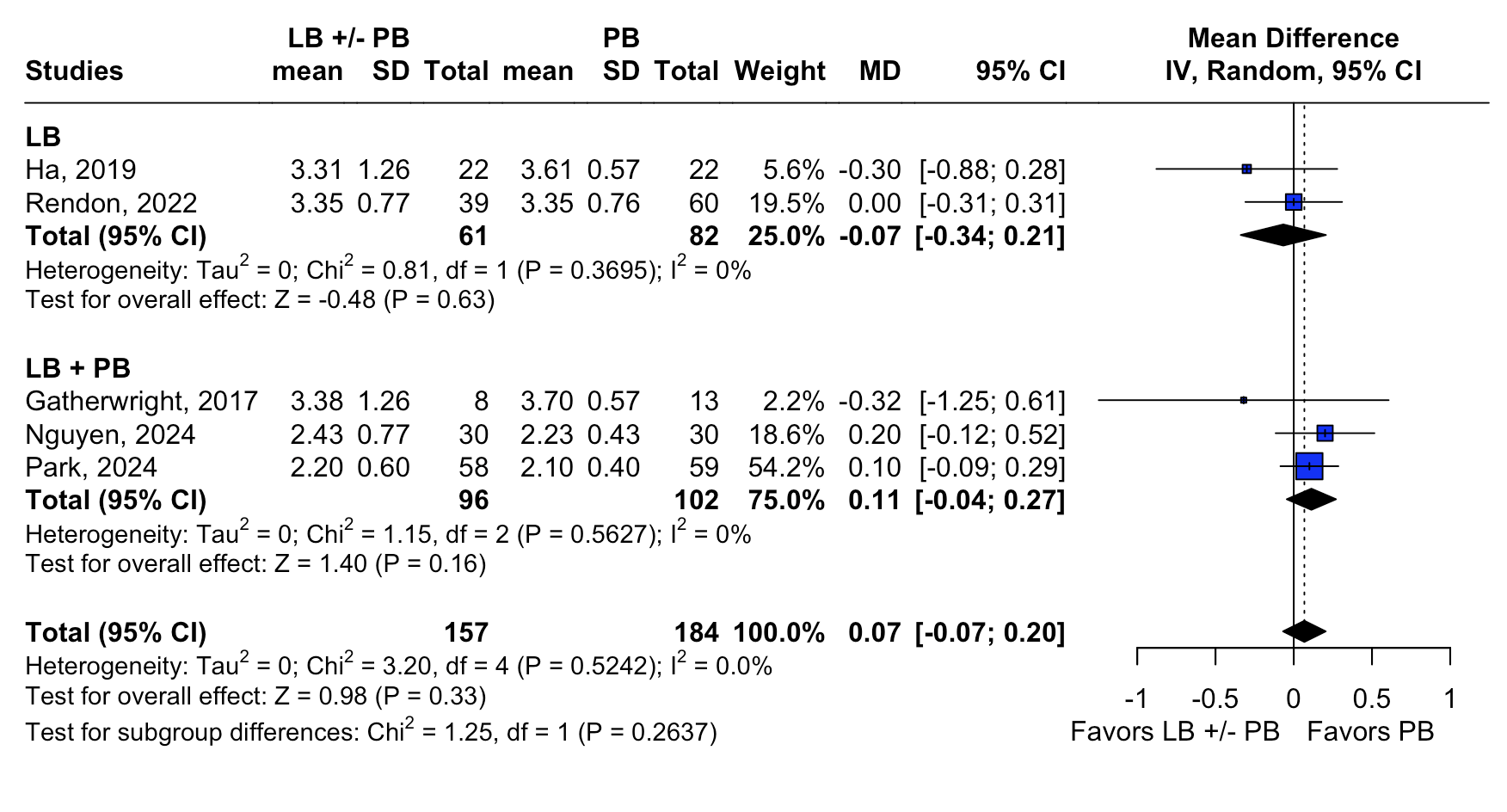

Supplement: Supplementary file 5 — Figure S5: micr70126‐sup‐0005‐FigureS5.png. [file MICR-45-e70126-s010.png]

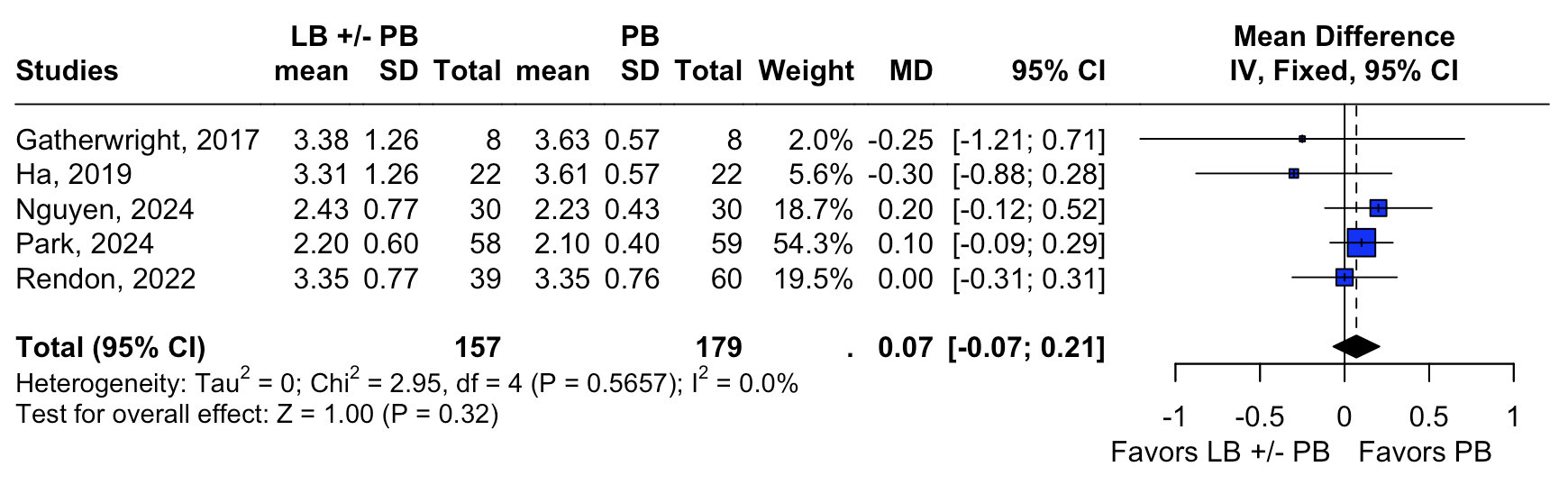

Supplement: Supplementary file 6 — Figure S6: micr70126‐sup‐0006‐FigureS6.png. [file MICR-45-e70126-s003.png]
