# Supplementary material for: Liposomal Bupivacaine in Transversus Abdominis Plane Block for Postoperative Pain Control After Autologous Breast Reconstruction: A Systematic Review and Meta‐Analysis
Source: Microsurgery. 2025 Oct 3;45(7):e70126. doi: 10.1002/micr.70126 (PMC12493009; doi:10.1002/micr.70126)
Supplement: Supplementary file 7 — Table S1: micr70126‐sup‐0007‐TableS1.docx. [file MICR-45-e70126-s002.docx]

**Supplementary Table 1.** *Search strategy*. Search strategy by database.

| **Database** | **Search strategy** |
| --- | --- |
| **Cochrane Library** | #1 MeSH descriptor: [Mammaplasty] explode all trees  #2 MeSH descriptor: [Bupivacaine] explode all trees  (#1 OR "deep inferior epigastric artery perforator flap" OR "deep inferior epigastric artery perforator flaps" OR "deep inferior epigastric perforator" OR "deep inferior epigastric perforator flaps" OR "DIEP flap" OR "DIEP flaps" OR "deep inferior epigastric perforator flap" OR "TRAM flap" OR "TRAM flaps" OR "transverse rectus abdominis musculocutaneous flaps" OR "transverse rectus abdominis myocutaneous flap" OR "transverse rectus abdominis myocutaneous flaps" OR "transverse rectus abdominis musculocutaneous flap" OR "breast reconstruction" OR "breast plastic operation" OR "cosmetic breast operation" OR "mammaplasty" OR "mammoplast" OR "mammoplasty" OR "mastoplasty" OR "plastic operation, breast" OR "breast reconstruction") AND (#2 OR "1 butyl 2', 6' pipecoloxylidide" OR "1 butyl n (2, 6 dimethylphenyl) 2 piperidinecarboxamide" OR "1 butyl n (2, 6 dimethylphenyl) piperidine 2 carboxamide" OR "ah 2250" OR "ah2250" OR "anekain" OR "bicain" OR "bipuvacaine" OR "bucain" OR "bucaine" OR "bupi 25" OR "bupi25" OR "bupicain" OR "bupicaina" OR "bupinex" OR "bupirop" OR "bupirop simple sin preservantes" OR "bupisen" OR "bupivacain" OR "bupivacaine carbonate" OR "bupivacaine heavy" OR "bupivacaine hydrochloride" OR "bupivacaine hydrochloride kit" OR "bupivacaine hydrochloride preservative free" OR "bupivacaine liposome" OR "bupivan" OR "bupivicaine" OR "bupizenge" OR "buvacaina" OR "buvacainas" OR "buvasin" OR "carbostesin" OR "chirocaina" OR "clx 117" OR "clx117" OR "dolanaest" OR "dur 843" OR "dur843" OR "eladur" OR "exparel" OR "exparel liposomal" OR "gtx 101" OR "gtx101" OR "inibsa" OR "kamacaine" OR "lac 43" OR "lac43" OR "liq 865" OR "liq865" OR "macaine" OR "marcain" OR "marcaina" OR "marcaine" OR "marcaine hcl" OR "marcaine hydrochloride" OR "marcaine hydrochloride preservative free" OR "marcaine plain" OR "marcaine spinal" OR "optesia" OR "picain" OR "posidur" OR "posimir" OR "senpivac" OR "sensocaine" OR "sensorcaine" OR "sensorcaine-mpf" OR "sensoricaine" OR "sky 0302" OR "sky 0402" OR "sky0302" OR "sky0402" OR "win 11318" OR "win11318" OR "xaracoll" OR "bupivacaine") |
| **Embase** | 'bupivacaine'/exp AND ('breast reconstruction'/exp OR 'deep inferior epigastric perforator flap'/exp OR 'transverse rectus abdominis musculocutaneous flap'/exp) |
| **PubMed** | ("deep inferior epigastric artery perforator flap" OR "deep inferior epigastric artery perforator flaps" OR "deep inferior epigastric perforator" OR "deep inferior epigastric perforator flaps" OR "DIEP flap" OR "DIEP flaps" OR "deep inferior epigastric perforator flap" OR "TRAM flap" OR "TRAM flaps" OR "transverse rectus abdominis musculocutaneous flaps" OR "transverse rectus abdominis myocutaneous flap" OR "transverse rectus abdominis myocutaneous flaps" OR "transverse rectus abdominis musculocutaneous flap" OR "breast surgery" OR "breast reconstruction" OR "breast plastic operation" OR "cosmetic breast operation" OR "mammaplasty" OR "mammoplast" OR "mammoplasty" OR "mastoplasty" OR "plastic operation, breast" OR "breast reconstruction") AND ("1 butyl 2', 6' pipecoloxylidide" OR "1 butyl n (2, 6 dimethylphenyl) 2 piperidinecarboxamide" OR "1 butyl n (2, 6 dimethylphenyl) piperidine 2 carboxamide" OR "ah 2250" OR "ah2250" OR "anekain" OR "bicain" OR "bipuvacaine" OR "bucain" OR "bucaine" OR "bupi 25" OR "bupi25" OR "bupicain" OR "bupicaina" OR "bupinex" OR "bupirop" OR "bupirop simple sin preservantes" OR "bupisen" OR "bupivacain" OR "bupivacaine carbonate" OR "bupivacaine heavy" OR "bupivacaine hydrochloride" OR "bupivacaine hydrochloride kit" OR "bupivacaine hydrochloride preservative free" OR "bupivacaine liposome" OR "bupivan" OR "bupivicaine" OR "bupizenge" OR "buvacaina" OR "buvacainas" OR "buvasin" OR "carbostesin" OR "chirocaina" OR "clx 117" OR "clx117" OR "dolanaest" OR "dur 843" OR "dur843" OR "eladur" OR "exparel" OR "exparel liposomal" OR "gtx 101" OR "gtx101" OR "inibsa" OR "kamacaine" OR "lac 43" OR "lac43" OR "liq 865" OR "liq865" OR "macaine" OR "marcain" OR "marcaina" OR "marcaine" OR "marcaine hcl" OR "marcaine hydrochloride" OR "marcaine hydrochloride preservative free" OR "marcaine plain" OR "marcaine spinal" OR "optesia" OR "picain" OR "posidur" OR "posimir" OR "senpivac" OR "sensocaine" OR "sensorcaine" OR "sensorcaine-mpf" OR "sensoricaine" OR "sky 0302" OR "sky 0402" OR "sky0302" OR "sky0402" OR "win 11318" OR "win11318" OR "xaracoll" OR "bupivacaine") |
| **Web of Science** | (ALL=(deep inferior epigastric artery perforator flap) OR ALL=(deep inferior epigastric artery perforator flaps) OR ALL=(deep inferior epigastric perforator) OR ALL=(deep inferior epigastric perforator flaps) OR ALL=(DIEP flap) OR ALL=(DIEP flaps) OR ALL=(deep inferior epigastric perforator flap) OR ALL=(TRAM flap) OR ALL=(TRAM flaps) OR ALL=(transverse rectus abdominis musculocutaneous flaps) OR ALL=(transverse rectus abdominis myocutaneous flap) OR ALL=(transverse rectus abdominis myocutaneous flaps) OR ALL=(transverse rectus abdominis musculocutaneous flap) OR ALL=(breast reconstruction) OR ALL=(mammaplasty) OR ALL=(mammoplasty) OR ALL=(mastoplasty) OR ALL=(breast reconstruction)) AND (ALL=(bupivacain) OR ALL=(bupivacaine carbonate) OR ALL=(bupivacaine hydrochloride) OR ALL=(bupivacaine liposome) OR ALL=(bupivicaine) OR ALL=(marcain) OR ALL=(marcaine) OR ALL=(marcaine hydrochloride) OR ALL=(sensorcaine) OR ALL=(bupivacaine)) |
