# Supplementary material for: Liposomal Bupivacaine in Transversus Abdominis Plane Block for Postoperative Pain Control After Autologous Breast Reconstruction: A Systematic Review and Meta‐Analysis
Source: Microsurgery. 2025 Oct 3;45(7):e70126. doi: 10.1002/micr.70126 (PMC12493009; doi:10.1002/micr.70126)
Supplement: Supplementary file 8 — Table S2: micr70126‐sup‐0008‐TableS2.docx. [file MICR-45-e70126-s007.docx]

**Supplementary Table 2.** *Baseline characteristics*. Main baseline participant characteristics of each included study (liposomal bupivacaine with or without plain bupivacaine / plain bupivacaine).

| **Study** | **Study sample sex** | **Age, mean ± SD** | **BMI**, **mean ± SD** | **Comorbidities (No., %)** | | | **Smoking status (No., %)** | | | **Oncology treatment (No., %)** | |
| --- | --- | --- | --- | --- | --- | --- | --- | --- | --- | --- | --- |
|  |  |  |  | **DM** | **HTN** | **CKD** | **Never** | **Former** | **Current** | **Radiotherapy** | **Chemotherapy** |
| Gatherwright et al. (2017)^17^ | Females | (1) 52.1; (2) 53.1; (3) 50;* | (1) 30; (2) 28; (3) 28;* | NA | NA | NA | NA | NA | NA | NA | NA |
| Ha et al. (2019)^11^ | Females | 49 ± 9.2 / 49 ± 10.0 | 29.1 ± 4.6 / 28.1 ± 4.5 | 1 (4.5) / 0 (0) | 3 (13.6) / 2 (9.1) | 1 (4.5) / 0 (0) | NA | NA | NA | 12 (54.5) / 15 (68.2) | 17 (77.3) / 17 (77.3) |
| Jablonka et al. (2017)^16^ | Females | 50.2 ± 8.5 / 50.6 ± 8.8 | 28.0 ± 5.4 / 26.2 ± 5.0 | NA | NA | NA | NA | NA | NA | 12 (30) /14 (29.2) | 7 (17.5) / 10 (20.8) |
| Nguyen et al. (2024)^13^ | Females | 53.0 ± 9.5 / 52.2 ± 9.8 | 29.6 ± 5.3 / 30.2 ± 4.3 | 8 (27) / 6 (21) | 9 (31) / 10 (34) | 0 (0) / 0 (0) | 12 (40) / 17 (57) | 5 (17) / 3 (10) | 13 (43) / 10 (33) | 6 (20) / 10 (33) | 10 (33) / 10 (33) |
| Park et al. (2024)^14^ | Females | 51.1 ± 8.7 / 51.9 ± 10.5 | 30.0 ± 6.3 / 31.6 ± 6.7 | 5 (8.6) / 8 (13.6) | NA | 0 (0) / 1 (1.7) | 41 (70.7) / 40 (67.8) | 14 (24.1) / 14 (23.7) | 4 (6.9) / 4 (6.8) | 16 (27.6) / (22.0) | 19 (32.8) / 11 (18.6) |
| Rendon et al. (2022)^10^ | Females | 54.9 ± 8.9 / 50.3 ± 10.4 | 31.3 ± 5.0 / 30.1 ± 5.7 | 4 (10) / 4 (7) | 15 (39) / 15 (25) | NA | 26 (67) / 42 (70) | 13 (33) / 15 (25) | 0 (0) / 3 (5) | NA | NA |

*Gatherwright et al. (2017) presented three subgroups: (1) liposomal bupivacaine transversus abdominis plane block, (2) plain bupivacaine transversus abdominis plane block, and (3) continuous bupivacaine transversus abdominis plane infusion.

BMI: body mass index; CKD: chronic kidney disease; DM: diabetes mellitus; HTN: hypertension NA: not available/not applicable; SD: standard deviation
