# Supplementary material for: Liposomal Bupivacaine in Transversus Abdominis Plane Block for Postoperative Pain Control After Autologous Breast Reconstruction: A Systematic Review and Meta‐Analysis
Source: Microsurgery. 2025 Oct 3;45(7):e70126. doi: 10.1002/micr.70126 (PMC12493009; doi:10.1002/micr.70126)
Supplement: Supplementary file 9 — Table S3: micr70126‐sup‐0009‐TableS3.docx. [file MICR-45-e70126-s004.docx]

**Supplementary Table 3.** *Surgical aspects*. Surgical characteristics of each included study (liposomal bupivacaine with or without plain bupivacaine / plain bupivacaine).

| **Study** | **Laterality (No., %)** | | **Timing (No., %)** | | **Abdominal flap (No.)** | | | | **Length, ± SD** | **Complications (No., %)** |
| --- | --- | --- | --- | --- | --- | --- | --- | --- | --- | --- |
|  | **Unilateral** | **Bilateral** | **Immediate** | **Delayed** | **DIEP** | **MS-TRAM** | **SIEA** | **TRAM** |  |  |
| Gatherwright et al. (2017)^17^ | 8 (100) / 13 (100) | 0 (0) / 0 (0) | 0 (0) / 0 (0) | 8 (100) / 13 (100) | NA | NA | NA | NA | NA | NA |
| Ha et al. (2019)^11^ | 8 (36.3) / 8 (36.3) | 14 (63.6) / 14 (63.6) | 3 (13.6) / 5 (22.7) | 19 (86.4) / 17 (77.3) | 24/25 | 9/10 | 3/0 | 0/2 | Mean anesthesia duration (h): 9.4 ± 1.8 / 9.4 ± 1.6 | **Seroma: 0 (0) / 0 (0)**  **Hematoma: 1 (4.5) / 0 (0)**  **Partial flap loss: 1 (4.5) / 0 (0)**  Abdominal cellulitis: 1 (4.5) / 1 (4.5) |
| Jablonka et al. (2017)^16^ | 18 (45) / 30 (62.5) | 22 (55) / 18 (37.5) | 33 (82.5) / 41 (85.4) | 7 (17.5) / 7 (14.6) | 55/62 | NA | 2/1 | 5/3 | NA | Major complications: 3 (7.5) / 6 (12.5)  **30-day readmission: 1 (2.5) / 3 (6.25)**  **Return to OR: 3 (7.5) / 7 (14.6)**  **Flap loss: 2 (5) / 2 (4.2)**  Transfusions: 1 (2.5) / 11 (22.9)  Other: 1 (2.5) / 5 (10.4) |
| Nguyen et al. (2024)^13^ | 9 (30) / 7 (23) | 21 (70) / 23 (77) | 5 (17) / 6 (20) | 25 (83) / 24 (80) | NA | NA | NA | NA | Mean operative time (min): 492 ± 118 / 504 ± 77 | In both groups, there were no narcotic complications, urinary retention, or flap loss. |
| Park et al. (2024)^14^ | 30 (51.7) / 25 (42.4) | 29 (50) / 34 (57.6) | 32 (55.2) / 28 (47.5) | 27 (46.5) / 31 (52.5) | NA | NA | NA | NA | Operating room time (min): 318.7 ± 90.1 / 319.9 ± 98.9 | Wound dehiscence: 7 (12.1) / 8 (13.6)  Wound infection: 0 (0) / 1 (1.7)  **Return to OR: 4 (6.9) / 5 (8.5)**  Medical complication: 3 (5.2) / 3 (5.1)  **Readmission: 2 (3.4) / 1 (1.7)**  **Seroma: 0 (0) / 1 (1.7)**  **Hematoma: 2 (3.4) / 2 (3.4)**  Venous thrombosis: 1 (1.7) / 4 (6.8)  Arterial thrombosis: 0 (0) / 1 (1.7)  **Flap failure: 1 (1.7) / 1 (1.7)** |
| Rendon et al. (2022)^10^ | 19 (49) / 38 (63) | 20 (51) / 22 (37) | 25 (64) / 42 (70) | 14 (36) / 18 (30) | NA | NA | NA | NA | Immediate (h): 10.8 ± 1.7 / 10.0 ± 1.9  Delayed (h): 11.2 ± 1.9 / 10.8 ± 2.4 | NA |

DIEP: Deep Inferior Epigastric Perforator; MS-TRAM: muscle-sparing transverse rectus abdominis myocutaneous; NA: not available/not applicable; OR: operating room; SD: standard deviation; SIEA: superficial inferior epigastric artery; TRAM: transverse rectus abdominis myocutaneous
