# Supplementary material for: Liposomal Bupivacaine in Transversus Abdominis Plane Block for Postoperative Pain Control After Autologous Breast Reconstruction: A Systematic Review and Meta‐Analysis
Source: Microsurgery. 2025 Oct 3;45(7):e70126. doi: 10.1002/micr.70126 (PMC12493009; doi:10.1002/micr.70126)
Supplement: Supplementary file 10 — Table S4: micr70126‐sup‐0010‐TableS4.docx. [file MICR-45-e70126-s008.docx]

**Supplementary Table 4.** *Interventions and perioperative protocols*. Detailed interventions and perioperative protocols adopted in each study.

| **Study** | **Intervention(s)** | **Perioperative protocols** |
| --- | --- | --- |
| Gatherwright et al. (2017)^17^ | (1) 266 mg LB + 60 mL with 20 mL of 0.25% PB; (2) 2 mg/kg of 0.25 % PB; (3) On-Q pump placed by means of ultrasound guidance in TAP block plane and run at a rate of 0.25% PB at 4 mL/hour (0.01g/hour) | NA |
| Ha et al. (2019)^11^ | 266 mg LB vs. 75 mg PB | **Preoperative Analgesia:** Paravertebral block (15 mL of 0.5% bupivacaine per side, T2–T4), oxycodone (OxyContin), and gabapentin.  **Postoperative Analgesia: *Day 0*,** IV hydromorphone (1 mg/hour as needed) ***Day 1 onward*,** Oral oxycodone (5–10 mg every 3 hours) + IV hydromorphone (0.5 mg/hour as needed).  **Nausea Control:** Scopolamine patch, intraoperative dexamethasone, ondansetron.  **Mobilization: *Day 1*,** Chair. ***Day 2*,** Assisted ambulation. ***Day 3*,** Independent ambulation. |
| Jablonka et al. (2017)^16^ | 20 mL of 1.3% LB + 30 mL of 0.25% PB + 80 mL of normal saline vs. 30 mL of 0.25% PB injected bilaterally, and then bilateral epidural catheters 0.25% PB (On-Q pump, 2 mL/h) | **Analgesia (both groups):**  *IV (Until Oral Intake Tolerated, ~Day 1):* Ketorolac 15 mg + Acetaminophen 1000 mg every 6 hours.  *Oral (After Day 1):* Ketorolac 10 mg + Acetaminophen 650 mg every 6 hours.  *Breakthrough Pain:* Oral opioids as needed.  *At Discharge:* Ketorolac 10 mg every 6 hours (until postoperative day 5) + Acetaminophen 650 mg every 6 hours as needed + Oral opioid if required.  **Thromboprophylaxis:**  ***TAP-Catheter Group:*** Heparin 5000 U SC every 12 hours (2 doses, discontinued after ambulation). Leg compression devices in bed. ***TAP-Liposomal Bupivacaine Group:*** Heparin 5000 U SC every 12 hours (2 doses). Leg compression devices in bed. Ketorolac continued for 5 days. |
| Nguyen et al. (2024)^13^ | 266 mg (20 mL) of 1.3% LB + 20 mL of 0.25% vs. 20 mL of 0.25% PB | **Preoperative:** Acetaminophen **1000 mg** + Gabapentin **300 mg** (or **100 mg** if >65 years), transdermal scopolamine patch.  **Postoperative Pain Management:** *Scheduled -* Celecoxib 100–200 mg every 12 hours, acetaminophen 1000 mg every 8 hours, gabapentin 300 mg every 8 hours (or 100 mg if >65 years). *As Needed* - Cyclobenzaprine 5 mg every 8 hours, oxycodone 5 mg every 4 hours or Tramadol 50 mg every 8 hours.  **Nausea Control:** *Preemptive* - Scopolamine patch. *As Needed* - Ondansetron 4 mg every 6 hours.  **Postoperative Day 1:** Regular diet initiated. Foley catheter removed. Assisted ambulation.  **Discharge (Typically at 48 Hours):** Same oral pain regimen continued (except celecoxib, unless already home medication). Encouraged to take pain medication as needed. |
| Park et al. (2024)^14^ | 30 mL of 0.25% PB, 0.15 mL of 1:1000 epinephrine, and 50 mL of normal saline, and 20 mL of LB (266 mg) vs. 30 mL of 0.25% PB, 0.15 mL of 1:1000 epinephrine, and 50 mL of normal saline | **Preoperative:** *Erector Spinae Plane (ESP) Blocks* - ≥70 kg: 20 mL of 0.25% plain bupivacaine per side; <70 kg: 15 mL of 0.25% plain bupivacaine per side. *Anesthesia* - Total intravenous anesthesia (TIVA) with propofol (100–150 μg/kg/min) + ketamine (0.15–0.30 mg/kg/h). Conservative fluid management.  **Postoperative Pain Management:** *Scheduled -* Acetaminophen 950 mg every 6 hours. IV ketorolac 30 mg every 8 hours (for 6 doses). After IV ketorolac: Ibuprofen 600 mg every 6 hours. *As Needed* - Oxycodone every 4 hours based on VAS pain score (4–6 (moderate pain): 5 mg; 7–10 (severe pain): 10 mg) Nurses assess pain every 2–4 hours, especially around medication times.  **Catheter Removal:** *Case duration <5 hours* - Foley removed at end of surgery. *Case duration ≥5 hours* - Foley removed morning of POD 1.  **Mobilization & Discharge:** *POD 1*, Physical & occupational therapy started. *POD 2*, Patients discharged home if stable.  **Discharge Medications:** Continue acetaminophen + ibuprofen for 1 week; 15 tablets of 5 mg oxycodone provided for breakthrough pain. |
| Rendon et al. (2022)^10^ | 60 mL of a mixture containing 266 mg of LB + 120 mL saline injected as two equal doses into each TAP plane vs. 400 mL of 0.5% PB with an infusion of rate of 4 mL/h | **Preoperative:** Oxycodone 10 mg PO, Gabapentin 900 mg PO, Acetaminophen 975 mg PO, Scopolamine TD**Intraoperative:** Dexamethasone 4 mg IV, Ondansetron 4 mg IV, Ketamine 0.25 mg/kg/hour, Fentanyl < 2–4 mcg/kg, Avoid hydromorphone, Esophageal Doppler to assist fluid management, Normotension, normothermia, and normoglycemia**PACU:** On-Q pump / Liposomal Bupivacaine to abdominal incision, Promethazine 6.25 mg IV PRN, Haloperidol 1 mg IV PRN, Ketorolac 30 mg IV PRN, Oxycodone 5–10 mg PO PRN, Hydromorphone 0.2–0.5 mg IV PRN**Hospital floor:** Acetaminophen 650 mg PO TID × 6, Ibuprofen 800 mg PO TID × 6, Gabapentin 100 mg TID, Oxycodone 5–10 mg PO q4h PRN, Hydromorphone 0.2–0.5 mg IV q4h PRN; Early out-of-bed to chair and ambulation; Antibiotic prophylaxis and thromboprophylaxis per standard of care; Early discontinuation of catheters and IV lines |

LB: liposomal bupivacaine; NA: not available/not applicable; PB: plain bupivacaine; POD: postoperative day
